# Distribution and diversity of cultured endophytic fungi in *Gentiana straminea* Maxim. at different altitudes on the northeastern Qinghai-Tibetan Plateau

**DOI:** 10.3389/fmicb.2024.1466613

**Published:** 2024-10-24

**Authors:** Tingfeng Cheng, Pengcheng Lin, Dangwei Zhou, Huan Wang, Kun Zheng, Jianwei Shen, Shengbo Shi, Xingqiang Hu, Xing Ye, Xueye Cao

**Affiliations:** ^1^Key Laboratory of Adaptation and Evolution of Plateau Biota (AEPB), Northwest Institute of Plateau Biology, Chinese Academy of Sciences, Xining, China; ^2^University of Chinese Academy of Sciences, Beijing, China; ^3^Key Laboratory of Engineering Biology for Low-carbon Manufacturing, Tianjin Institute of Industrial Biotechnology, Chinese Academy of Sciences, Tianjin, China; ^4^The College of Pharmacy, Qinghai Nationalities University, Xining, China; ^5^Key Laboratory for Tibet Plateau Phytochemistry of Qinghai Province, Xining, China; ^6^Tibetan Medicine Center, Northwest Institute of Plateau Biology, Chinese Academy of Sciences, Xining, China

**Keywords:** *Gentiana straminea* Maxim., endophytic fungi, altitudes, diversity, Qinghai-Tibetan Plateau

## Abstract

Endophytic fungi are a crucial microbial resource that can influence plant growth and development through their interactions with host plants. *Gentiana straminea* Maxim. is an important traditional Tibetan herb used to treat a range of diseases in the Qinghai-Tibetan region. However, the diversity and community structure of endophytic fungi in the species remain poorly understood. In this study, a total of 944 strains of endophytic fungi were isolated from the roots, stems, and leaves of *G. straminea* from four different altitudes. A total of 87 OTUs were identified through sequence alignment, comprising 6 classes, 15 orders, 25 families, and 44 genera. The colonization rate and diversity of endophytic fungi were affected by tissue type and altitude. With the exception of Xining, the endophytic fungi colonization rate of tissues was roots>leaves>stems. Moreover, the *α*-diversity of endophytic fungi among different tissues was leaves>stems>roots. Notably, the phylogenetic diversity index in leaves was significantly higher than that in roots. In addition, the colonization rate and diversity of endophytic fungi in leaves and stems demonstrated a decline with the increasing altitude. The *β*-diversity analysis revealed significant differences in the endophytic fungi of *G. straminea* at varying altitudes. In roots, geographical factors, such as latitude and longitude, were the primary drivers of variation, whereas environmental factors, including temperature and precipitation, had a greater influence on endophytes in leaves and stems. In addition, the results of the endophytic fungi association preference, linear discriminant analysis effect size (LEfSe), and co-network analysis indicated that these differential endophytic fungi may play a significant role in the authenticity and stress resistance of *G. straminea*.

## 1 Introduction

*Gentiana straminea* Maxim., commonly referred to as “Mahua Jiao,” is a traditional Chinese medicinal herb that belongs to the Sect. *Cruciata* Gaudin of *Gentiana* genus. It is primarily distributed across the Qinghai-Tibetan Plateau (Ho and Liu, 2001). The dried roots of *G. straminea*, *G. macrophylla* Pall., *G. dahurica* Fisch., and *G. crassicaulis* Duthie ex Burk. are classified as the original species of “Qinjiao” in the Chinese Pharmacopeia (Chinese Pharmacopoeia Commission, 2015). The species is abundant in iridoids, including gentiopicroside, loganic acid, and sweroside. These compounds may contribute to anti-inflammatory (Jia et al., 2012; Jia et al., 2016; Wang et al., 2020), hepatoprotective (Dai et al., 2018), gastrointestinal protection (Ruan et al., 2015; Yang et al., 2018), cardioprotective (Huang et al., 2015), and antifungal action (Tan et al., 1996).

Endophytic fungi have been identified in almost all plants and have the potential to produce a variety of bioactive compounds, including terpenoids, phenols, and alkaloids. These substances could serve as valuable alternative sources of medicinal compounds (Toghueo and Boyom, 2020; Torres Mendoza et al., 2020). Meanwhile, additional research has revealed that endophytic fungi possess a multitude of biological functions, which can enhance the stress tolerance of host plants and stimulate their growth through diverse mechanisms (Yang et al., 2018). In particular, their secondary metabolites have been demonstrated to possess a range of biological activities, including antibacterial, antifungal, insecticidal, herbicidal, cytotoxic, antioxidant, and anticancer effects (Gupta et al., 2020; Xie et al., 2020).

The Qinghai-Tibet Plateau is known as the “Roof of the World” and the “Third Pole.” The extreme environmental conditions that are unique to the plateau, including low oxygen, low air pressure, low temperature, and strong ultraviolet radiation, exert a significant influence on microorganisms (Zheng and Zhao, 2017). The paucity of studies on endophytic fungi in the Qinghai-Tibet Plateau is largely attributable to the limitations of sampling conditions and the vast geographical area (Harrison and Griffin, 2020). A substantial body of research has been conducted on *G. straminea*, encompassing phytochemicals, pharmacology, and quality control (Pan et al., 2020; Li et al., 2020); however, there is a paucity of literature on the endophytic fungi associated with this species. The continued destruction of wild resources of *G. straminea* is a consequence of the high quality and high demand for this product in the market (Li et al., 2013). Accordingly, the objective of this study was to explore the diversity profiling of endophytic fungi and identify potential alternative resources. To this end, endophytic fungi were isolated from the roots, stems, and leaves of *G. straminea* at four distinct altitudes. The community structure, diversity, and relationship with environmental factors were elucidated and discussed, thus contributing to the development and utilization of endophytic fungi resources in the Qinghai-Tibet Plateau.

## 2 Materials and methods

### 2.1 Plant materials

*Gentiana straminea* samples were collected from four distinct elevation gradients in Qinghai during the flowering period (Supplementary Table 1). The voucher specimen was authenticated by Prof. Chen Shilong (Northwest Institute of Plateau Biology, Chinese Academy of Sciences).

### 2.2 Isolation and purification of endophytic fungi

Endophytic fungi were isolated by placing surface-sterilized tissue fragments (roots, stems, and leaves) on the culture medium (Zeng et al., 2015; Cheng et al., 2021). Plant samples were rinsed with tap water and then subjected to surface sterilization in the following manner: they were immersed in 75% ethanol for 1 min, followed by 2.5% sodium hypochlorite for 1 min, and then rinsed six times with sterile water. Subsequently, the sterilized samples were inoculated into potato dextrose agar (PDA medium) in Petri plates and cultured in a growth chamber in the dark at a constant temperature of 28°C. The plates were observed on a daily basis, and the appearance of fungal colonies was recorded as they emerged.

### 2.3 Identification of endophytic fungi

The macroscopic characteristics and the microscopic individuality of the endophytic fungi were recorded and classified using the lactic acid–phenol method, as described in the fungal identification manual (Wei, 1997). Genomic DNA was extracted from fungal mycelia using the cetyltrimethylammonium bromide (CTAB) method. The primers ITS1 and ITS4 were employed for the amplification of the ITS region of the fungal DNA (Guo et al., 2001). The amplifications were purified and sequenced by the Sangon Biotech (Shanghai) Co., Ltd. The MEGA6.5 software was employed to excise chimeric bases, thereby reducing the length of each sequence to approximately 550 bp. The Mothur program (Schloss et al., 2009) was employed for the identification of sequences and the grouping of consensus sequences into the same operational taxonomic units (OTUs), with a sequence identity threshold of 97%. A phylogenetic tree was constructed using the neighbor-joining method, as implemented in MEGA6.5 (Koichiro et al., 2013), with 1,000 bootstrap replications.

### 2.4 Endophytic fungi diversity analysis

The abundance and diversity of endophytic fungi in *G. straminea* were analyzed at the genus level. The isolation colonization rate, relative frequency, diversity index, and other indicators of endophytic fungi in different tissues of *G. straminea* at varying altitudes were calculated according to the method described previously (Kusari et al., 2013). The *α* diversity index is primarily concerned with the species richness and diversity of a given community (Whittaker, 1972; Shannon, 2001; Simpson, 1997). It encompasses a number of different indices, including Menhinick’s index (Dmn, species richness), Shannon-Wiener diversity index (H′, species diversity, mainly for advantages species), Gini-Simpson diversity index (1-D, species diversity, mainly for rare species), phylogenetic diversity (PD, evolutionary distance), and Pielou evenness index (E, species distribution uniformity) (Pielou, 1966). The *β* diversity index is primarily concerned with the dissimilarities in species composition between disparate communities. It encompasses a range of indices, including the Jaccard similarity index (commonly utilized to assess community or quadrat species similarity) and the Bray-Curtis distance (incorporating species abundance data) (Brglez et al., 2020; Legendre and De Caceres, 2013). The preferences of the host–fungus association were evaluated in accordance with the methodology outlined in the study conducted by Toju et al. (2016).

### 2.5 Statistical analyses

The Microsoft Excel 2010 software was utilized to collate and calculate the colonization data of endophytic fungi. Based on the OTUs abundance table, the “picante” and “vegan” packages in the R were used to calculate the *α* diversity index (diversity) and *β* diversity index (vegdist). Based on the Bray–Curtis distance, the “stats” package in the R language was used for hierarchical clustering (hclust). All results were presented in the “ggplot2” package and Prism 8 (GraphPad Software, Inc., San Diego, CA, USA).

## 3 Results

### 3.1 Isolation rate and classification of endophytic fungi from *Gentiana straminea*

**Figure 1:**
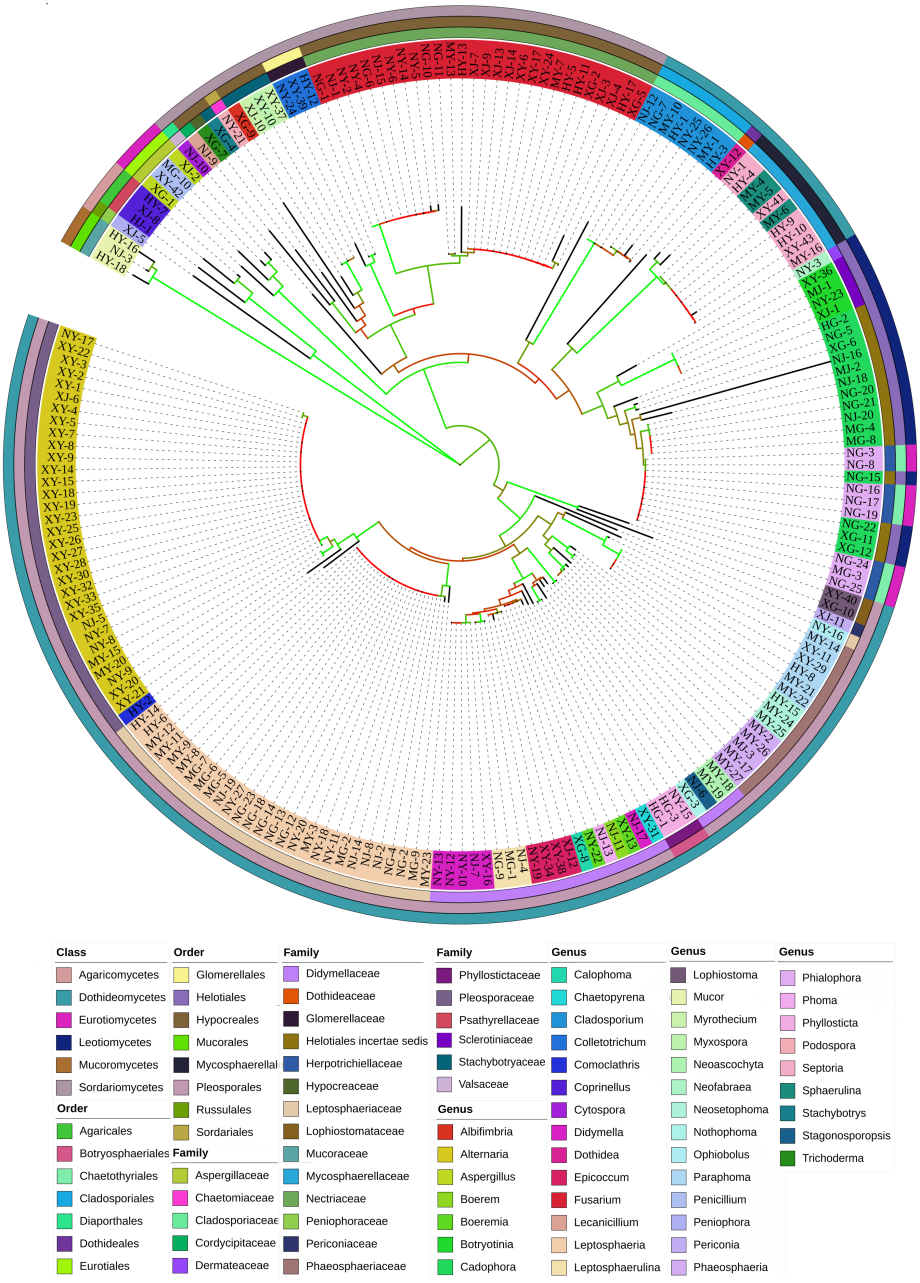
Phylogenetic tree of endophytic fungi from *G. straminea*.

A total of 944 strains of endophytic fungi were isolated from 1,968 tissue pieces of *G. straminea* at different altitudes. The strains were identified according to their colony morphological characteristics, resulting in the classification of 203 morphological species. The colony morphology and classification are illustrated in Supplementary Figure 1. In order to further identify the endophytic fungi, genomic DNA was extracted and used as a PCR template to amplify the ITS1-ITS4 region of 203 strains. The bands were found to correspond with the anticipated results and were sequenced using the Illumina MiSeq Sequencing platform. Operational taxonomic units (OTUs) are standardized markers employed in phylogenetic or population genetics research for classification (Supplementary Table 2). The isolated endophytic fungi encompassed a total of 6 classes, 15 orders, 25 families, and 44 genera (Figure 1).

### 3.2 Community structure and biodiversity of endophytic fungi from *Gentiana straminea*

#### 3.2.1 Colonization frequency

**Figure 2:**
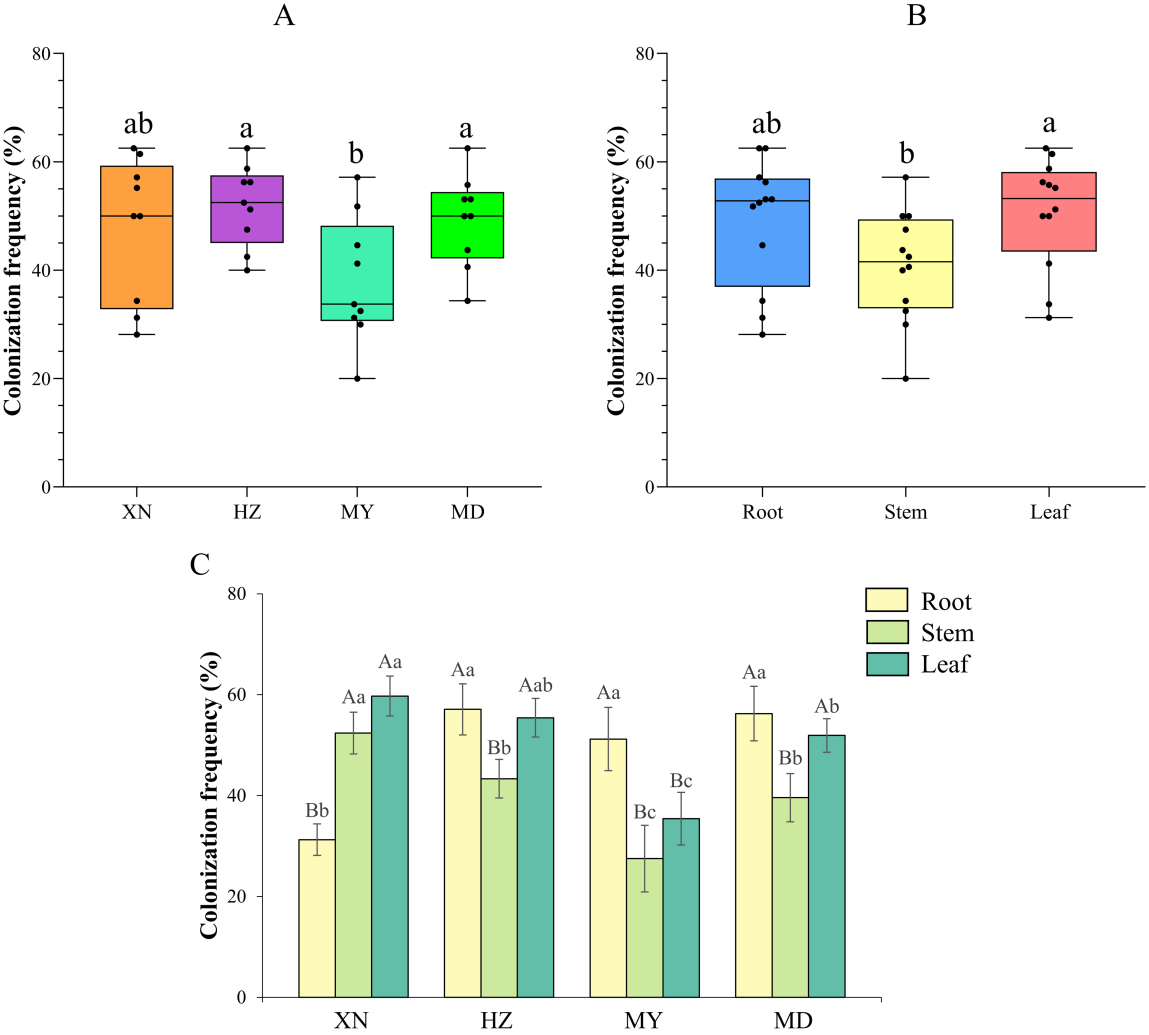
Colonization frequency of endophytic fungi from different tissues of *G. straminea* from different altitudes. **(A)** Colonization frequency of different altitudes; **(B)** Colonization frequency of different tissues; **(C)** Colonization frequency of different altitudes and tissues.

A total of 944 strains of endophytic fungi were obtained from the four locations: 246 from XN (Xining), 322 from HZ (Huzhu), 203 from MY (Menyuan), and 173 from MD (Maduo). The total colonization rates of endophytic fungi across the four altitudes were 51.94% in HZ, 49.25% in MD, 47.78% in XN, and 38.03% in MY. HZ and MD exhibited significantly higher colonization rates than MY (*p* < 0.05) (Figure 2A). Additionally, the colonization rate of endophytic fungi was found to be significantly higher in leaves than in stems (Figure 2B). In order to gain insight into the endophytic fungal colonization rate profiling in the tissues from different altitudes, the colonization rate was analyzed separately for each altitude. The results demonstrated that in the roots, HZ (57.08%) > MD (56.25%) > MY (51.19%) > XN (31.25%); in the stem, XN (52.38%) > HZ (43.33%) > MD (39.58%) > MY (27.50%); and in the leaves, XN (59.49%) > HZ (55.42%) > MD (51.92%) > MY (35.42%) (Figure 2C).

#### 3.2.2 Species composition and distribution

A total of 87 fungal taxa were identified based on morphological characteristics and DNA sequences of the nuclear internal transcribed spacer (ITS). The 39, 38, 25, and 24 OTUs were identified as belonging to the XN, HZ, MY, and MD groups, respectively. Furthermore, XN and HZ exhibited 17 distinct OTUs, while MY and MD demonstrated 13 distinct OTUs (Supplementary Figure 2). Notably, a single OTU was present in all samples collected from the different altitudes (Supplementary Figure 2A). Additionally, the tissues of the OTUs were subjected to analysis, which revealed the presence of 32, 32, and 63 OTUs in the root, stem, and leaves, respectively. A total of 11 OTUs were identified in all tissues (Supplementary Figure 2B).

**Figure 3:**
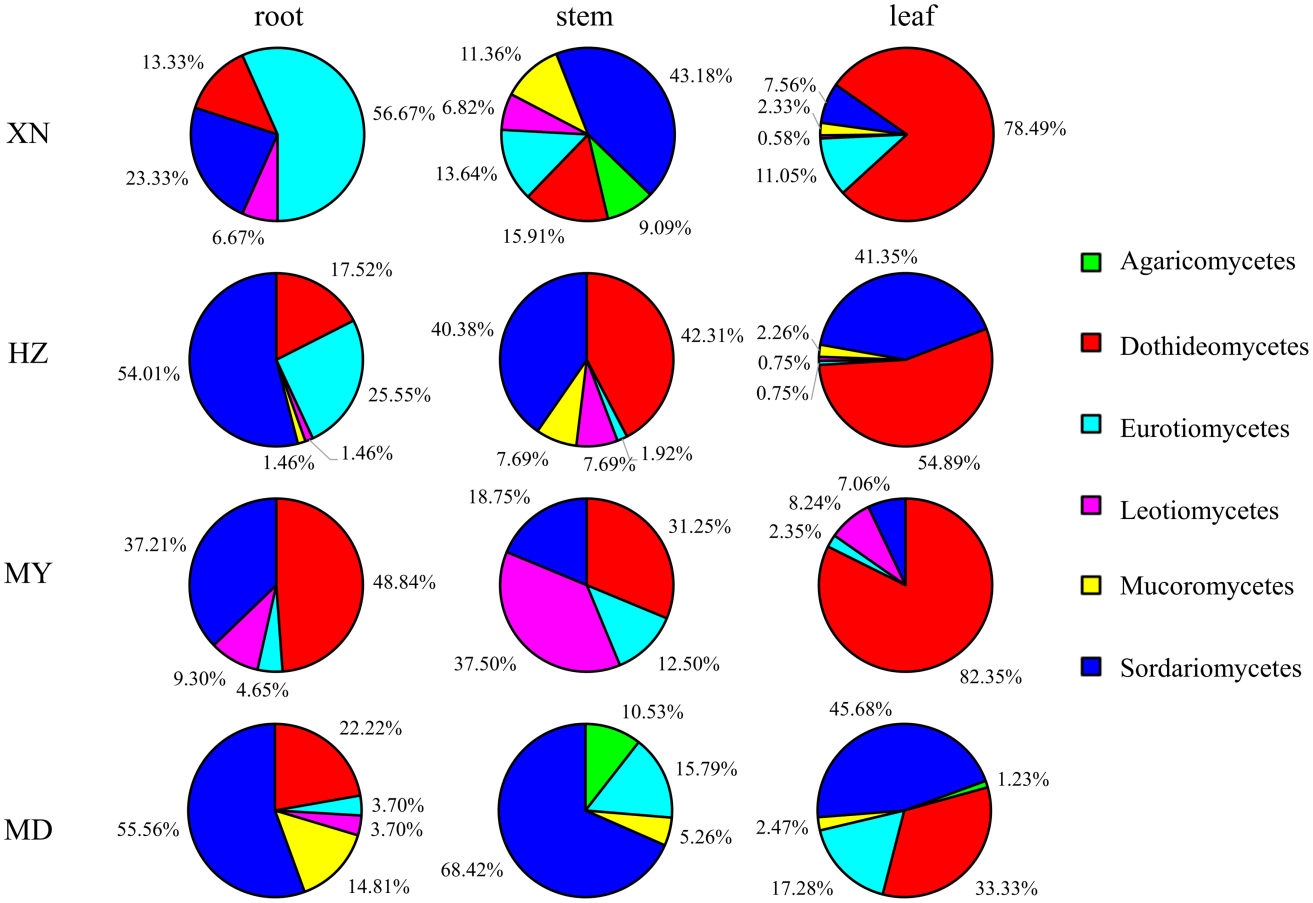
Relative frequency of endophytic fungi class from different tissues of *G. straminea* from different altitudes.

**Figure 4:**
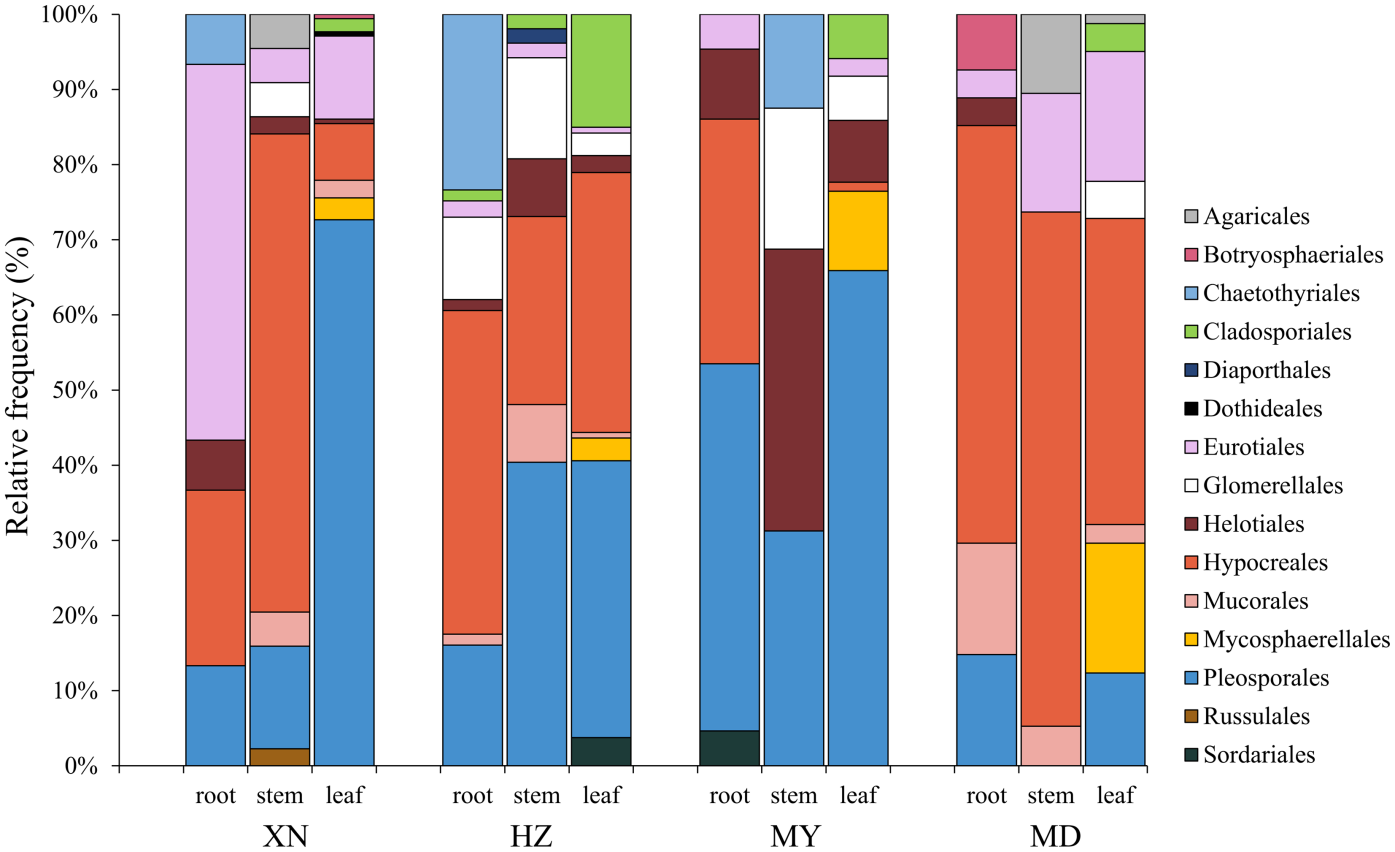
Relative frequency of endophytic fungi order from different tissues of *G. straminea* from different altitudes.

**Figure 5:**
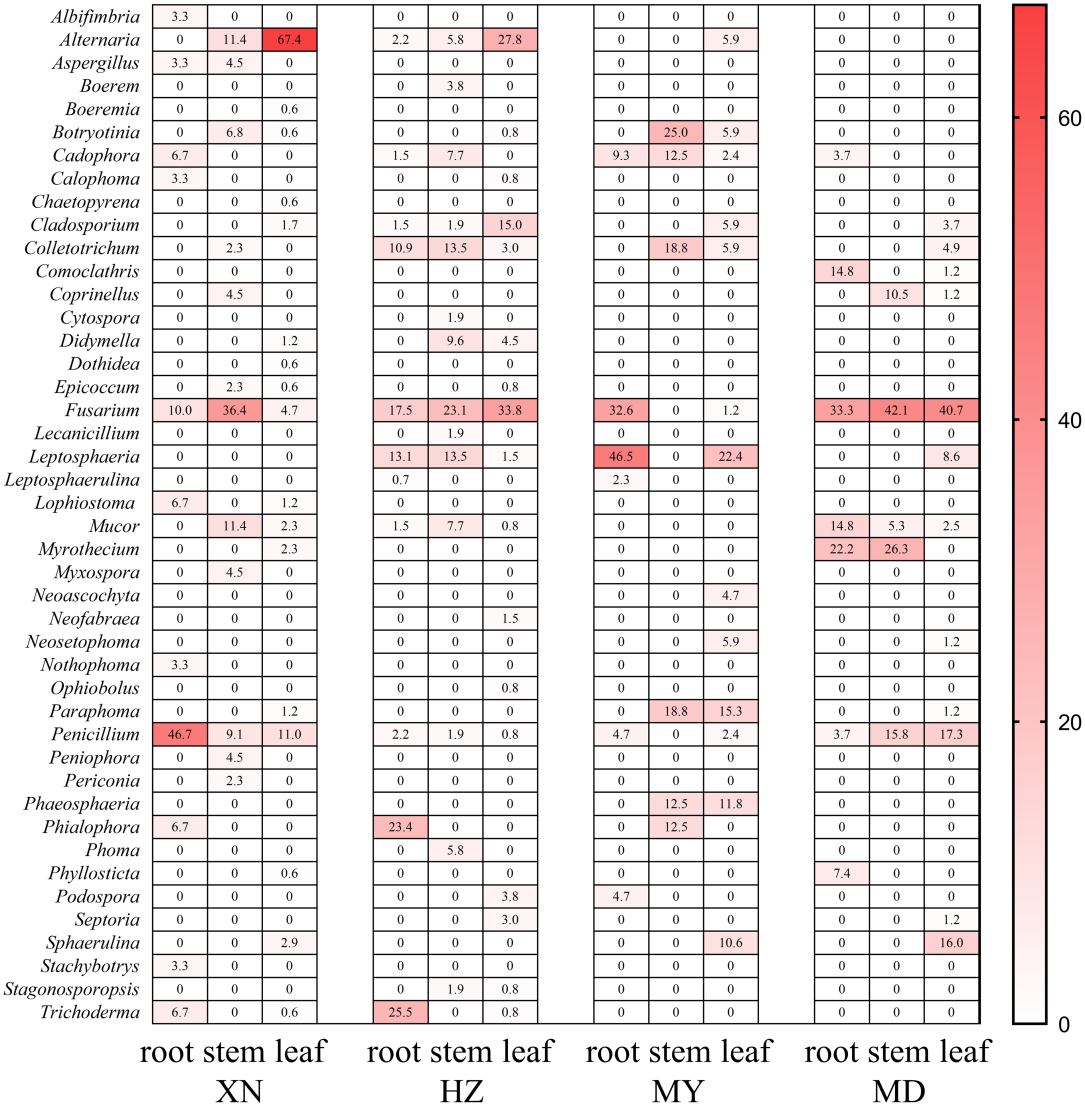
Relative frequency of endophytic fungi genus from different tissues of *G. straminea* from different altitudes.

The dominant classes were Dothideomycetes, Sordariomycetes, and Eurotiomycetes (Figure 3), and notable differences were observed among the altitudes. The prevalence of Sordariomycetes was observed to be higher in roots, stems, and leaves in MD than in other altitudes. Among the dominant classes, only Eurotiomycetes exhibited a decline in abundance with increasing altitude. The dominant orders were Pleosporales, Hypocreales, and Eurotiales (Figure 4). Moreover, the dominant genera were *Alternaria*, *Fusarium*, *Leptosphaeria*, *Penicillium*, *Trichoderma*, *Colletotrichum*, *Cladosporium*, *Cadophora*, *Phialophora*, *Mucor*, *Myrothecium*, *Paraphoma,* and *Sphaerulina* (Figure 5). In XN, the dominant genera in the roots, stems, and leaves were *Penicillium* (46.7%), *Fusarium* (36.4%), and *Alternaria* (67.4%), respectively. However, in MD, the dominant genus across all three tissues was *Fusarium* (Figure 5). *Alternaria* genera in leaves and *Penicillium* in roots exhibited a decline with increasing altitude, whereas *Sphaerulina* in leaves and *Fusarium* in roots demonstrated an increase with rising altitude.

#### 3.2.3 *α*-diversity analysis

**Figure 6:**
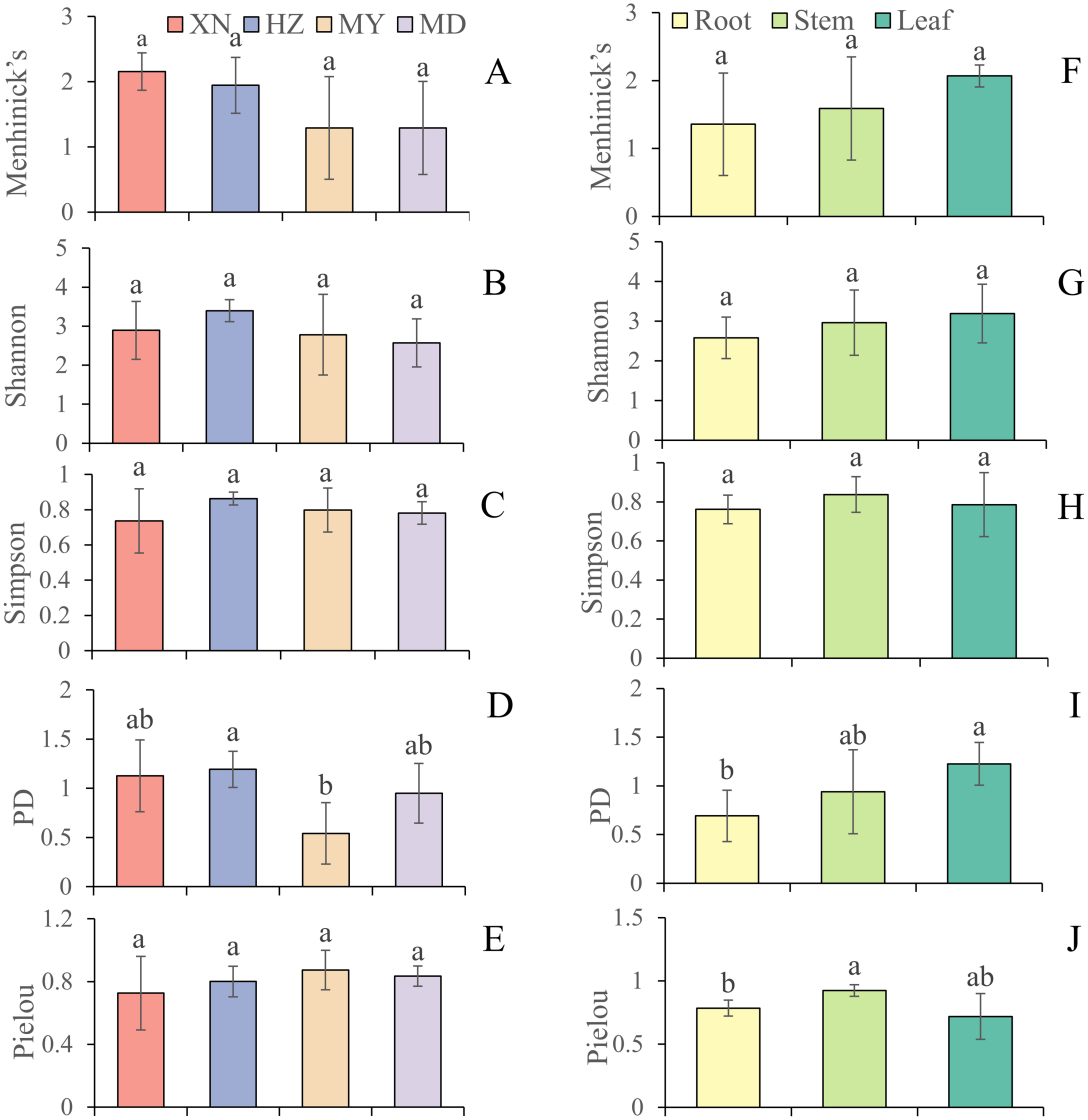
The diversity indices of endophytic fungi from different tissues of *G. straminea* from different altitudes. **(A)** Menhinick’s index of different altitudes; **(B)** Shannon index of different altitudes; **(C)** Simpson index of different altitudes; **(D)** Phylogenetic diversity index of different altitudes; **(E)** Pielou index of different altitudes; **(F)** Menhinick’s index of different tissues; **(G)** Shannon index of different tissues; **(H)** Simpson index of different tissues; **(I)** Phylogenetic diversity index of different tissues; **(J)** Pielou index of different tissues.

The species accumulation curves indicated that the observed fungal richness exhibited a continuous increase in all host plants, suggesting that further sampling would result in the recovery of additional endophytic taxa (Supplementary Figure 3). The diversity of endophytic fungi was ranked from highest to lowest as follows: HZ (H′:3.40, 1-D:0.86, PD:1.19), XN (H′:2.89, 1-D:0.74, PD: 1.12), MY (H′:2.78, 1-D:0.80, PD: 0.54), and MD (H′:1.29, 1-D:0.78, PD: 0.95). The PD in HZ was found to be significantly higher than in MY in whole plants (Figure 6; Supplementary Table 3). However, there is no significant differentiation evident among the XN, HZ, and MD samples. Moreover, the index of *α*-diversity exhibited variability across the three tissues. The diversity index demonstrated a decline with increasing altitude in the stems, while no such trend was observed in the roots and leaves. In the roots, an increased α-diversity was observed in XN and HZ, with MD exhibiting a moderately elevated diversity compared to MY. However, in the leaves, the highest index of α-diversity was observed in MY (H′:3.92, 1-D:0.91, PD:0.90). In contrast, the lowest index of α-diversity was observed in XN (H′:2.86, 1-D:0.76, PD:0.77) (Supplementary Table 3).

#### 3.2.4 *β*-diversity analysis

**Figure 7:**
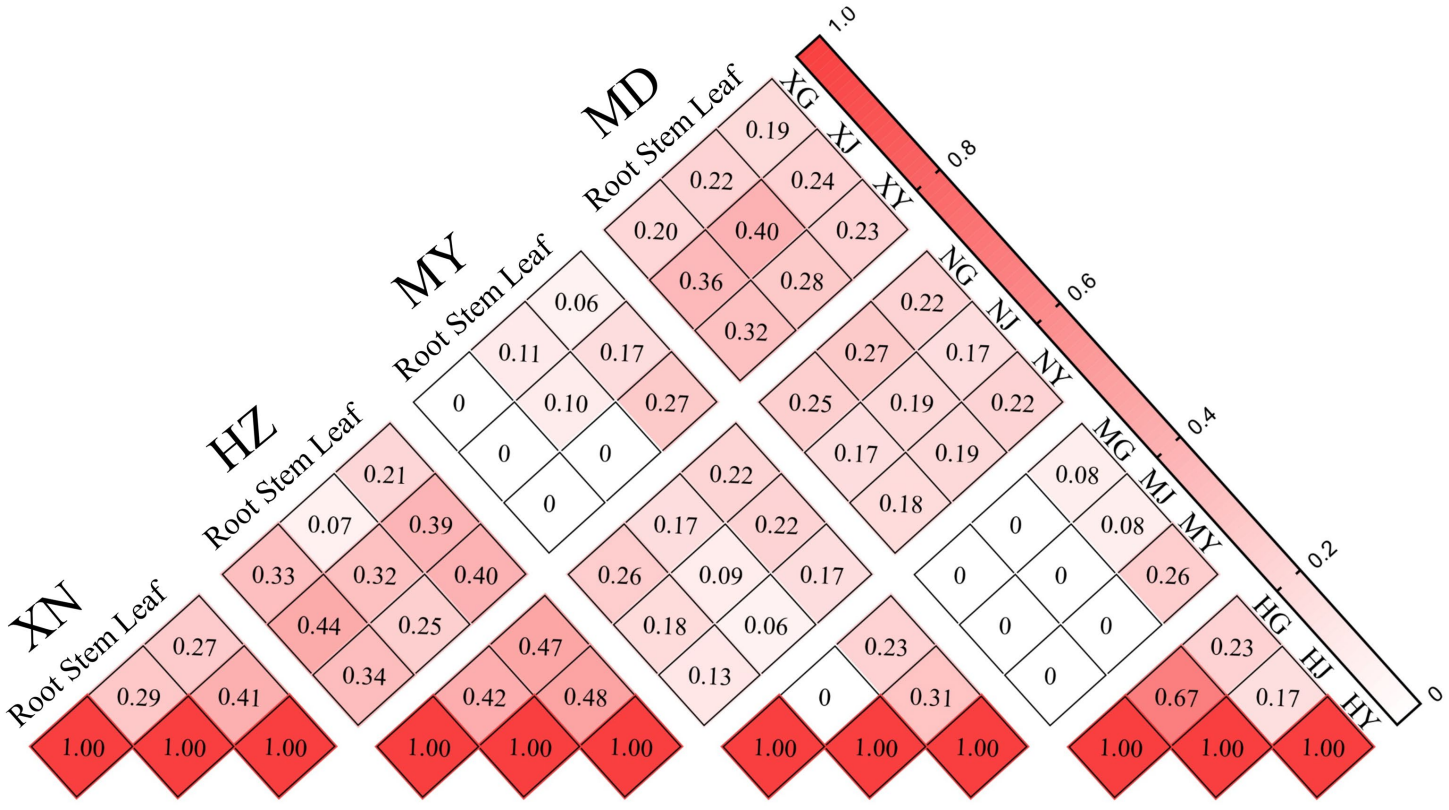
Similarity index of endophytic fungi from different tissues of *G. straminea* from different altitudes.

The Bray–Curtis similarity index revealed that the highest degree of similarity in the roots, stems, and leaves was observed between XN and HZ (D_Bray_: 0.33), XN and MD (D_Bray_: 0.40), and XN and HZ (D_Bray_: 0.40), respectively. The degree of similarity is high in the stems and leaves of XN (D_Bray_: 0.41), the stems and leaves of HZ (D_Bray_: 0.48), the stems and leaves of MY (D_Bray_: 0.31), and the roots and stems of MD (D_Bray_: 0.67). Furthermore, the lowest degree of similarity is observed for MY and XN, MY and MD of roots (D_Bray_:0), MY and MD of stems (D_Bray_:0), and MY and HZ of leaves (D_Bray_: 0.17). The roots and leaves of XN (D_Bray_: 0.27), the roots and stems of HZ (D_Bray_: 0.42), the roots and stems of MY (D_Bray_: 0), and the stems and leaves of MD (D_Bray_: 0.17) exhibited low similarity (Figure 7). Moreover, Jaccard’s similarity coefficient analysis revealed that XN and MD exhibited the highest similarity (C_j_: 0.262), while XN and MY demonstrated the lowest degree of similarity (C_j_: 0.02) (Supplementary Figure 4).

**Figure 8:**
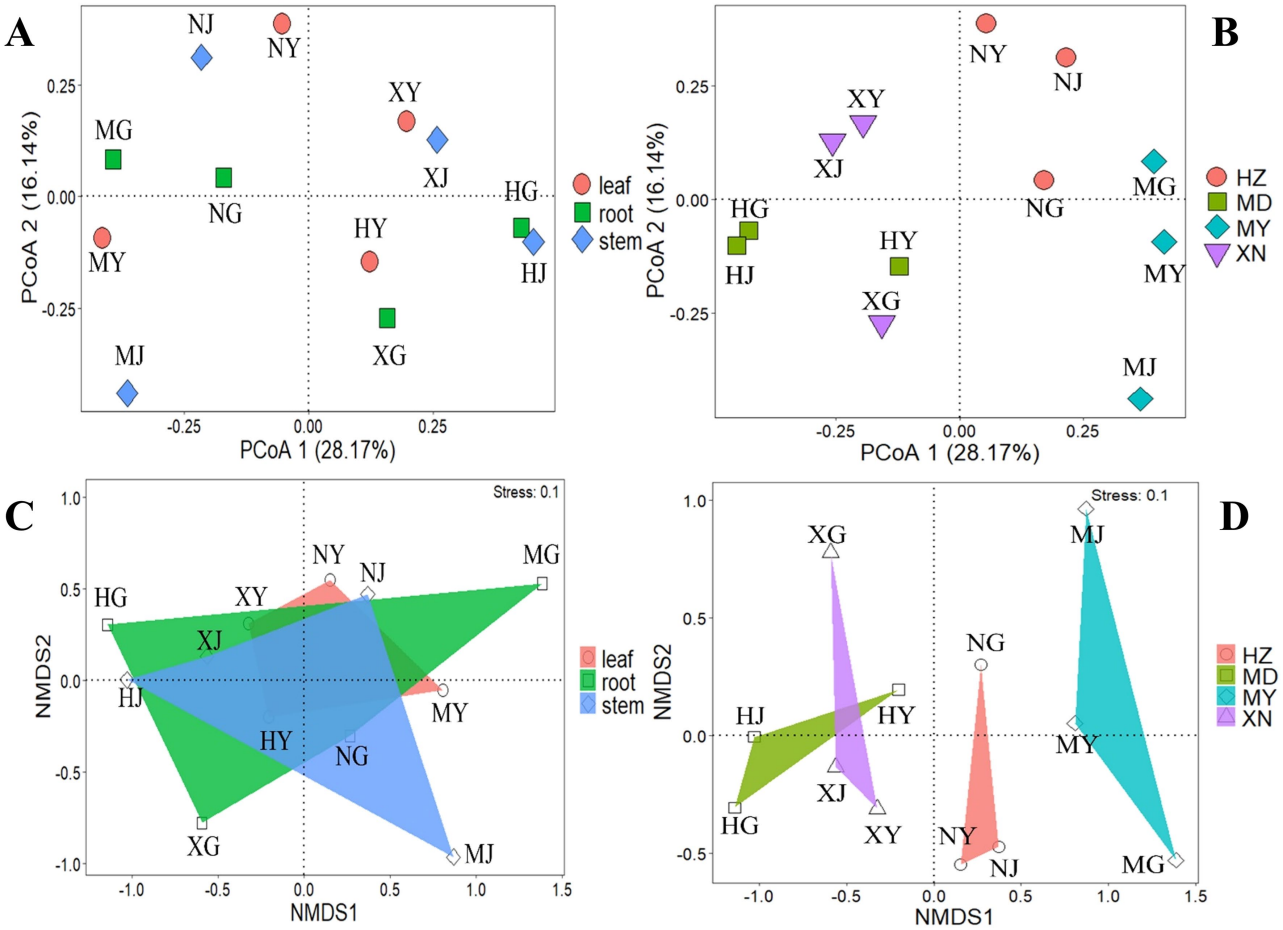
PCoA and NMDS analysis of endophytic fungi from different tissues of *G. straminea* from different altitudes. **(A)** PCoA of different tissues; **(B)** PCoA of different altitudes; **(C)** NMDS of different tissues; **(D)** NMDS of different altitudes.

In addition, the beta diversity patterns between the 12 samples were evaluated by plotting two-dimensional non-metric multidimensional scaling (NMDS) based on the relative abundance of the 87 OTUs. As illustrated in the cluster and NMDS plots, there were significant differences in fungal community composition among plant locations, rather than among the different tissues (Figure 8; Supplementary Figure 5).

### 3.3 Ordination analysis of endophytic fungi

**Figure 9:**
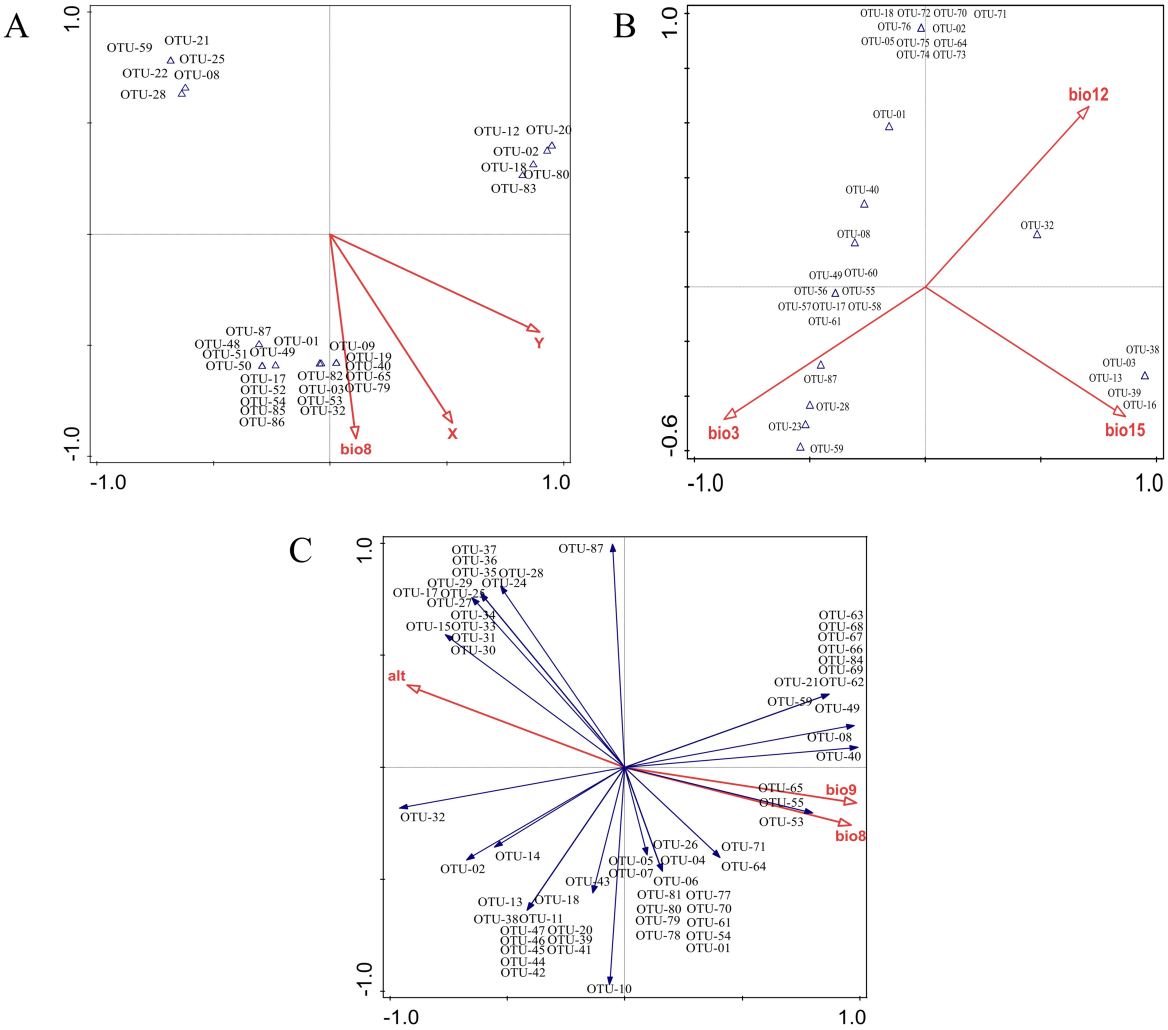
Ordination analysis of endophytic fungi from different tissues of *G. straminea*. **(A)** Canonical correspondence analysis of roots; **(B)** Canonical correspondence analysis of stems; **(C)** Redundancy analysis of leaves.

In order to comprehend the interconnection between ecological factors and the endophytic fungi population at the OTU level, an analysis of the OTUs-ecological factors was conducted using the canonical correspondence analysis (CCA) and redundancy analysis (RDA) models. Subsequent selection yielded 3 variations extracted from the 19 climatic and 3 environmental factors (Table 1). As illustrated in Figure 9, the primary ecological factors influencing the distribution of endophytic fungi within root systems were identified as x (longitude), y (latitude), and bio8 (mean temperature of wettest quarter https://www.worldclim.org/data/bioclim.html#google_vignette). These factors collectively accounted for 37.7, 35.3, and 26.8%, respectively. In stems, the endophytic fungi distribution was influenced by three bioclimatic variables: bio3 (isothermality), bio15 (precipitation seasonality), and bio12 (annual precipitation). These variables collectively explained 42.6, 36.6, and 20.8% of the ecological information, respectively. Similarly, the primary factors influencing the endophytic fungi of leaves were bio9 (mean temperature of driest quarter), altitudes, and bio8 (mean temperature of wettest quarter), which collectively explained 64.02, 20.9, and 15% of the ecological information, respectively.

**Table 1:** Conditional effects of variables on the forward selection of endophytic fungi from *G. straminea*.

Variables	Root			Stem			Leaf		
Ecological factors	Longitude (x)	Latitude (y)	bio8	bio3	bio15	bio12	bio9	altitude	bio8
Explanatory variable (%)	37.7	35.5	26.8	42.6	36.6	20.8	64.0	20.9	15.0
*p*-value	0.086	0.306	1.000	0.086	0.236	1.000	0.052	0.340	1.000
The first axis correlation	0.897	0.525	0.111	−0.872	0.866	0.708	0.986	−0.927	0.963
The secondary axis correlation	−0.442	−0.850	−0.919	−0.485	−0.475	0.661	−0.158	0.366	−0.258

bio3, Isothermality; bio8, Mean Temperature of Wettest Quarter; bio9, Mean Temperature of Driest Quarter; bio12, Annual Precipitation; bio15, Precipitation Seasonality.

### 3.4 Host–fungus association preferences of endophytic fungi

**Figure 10:**
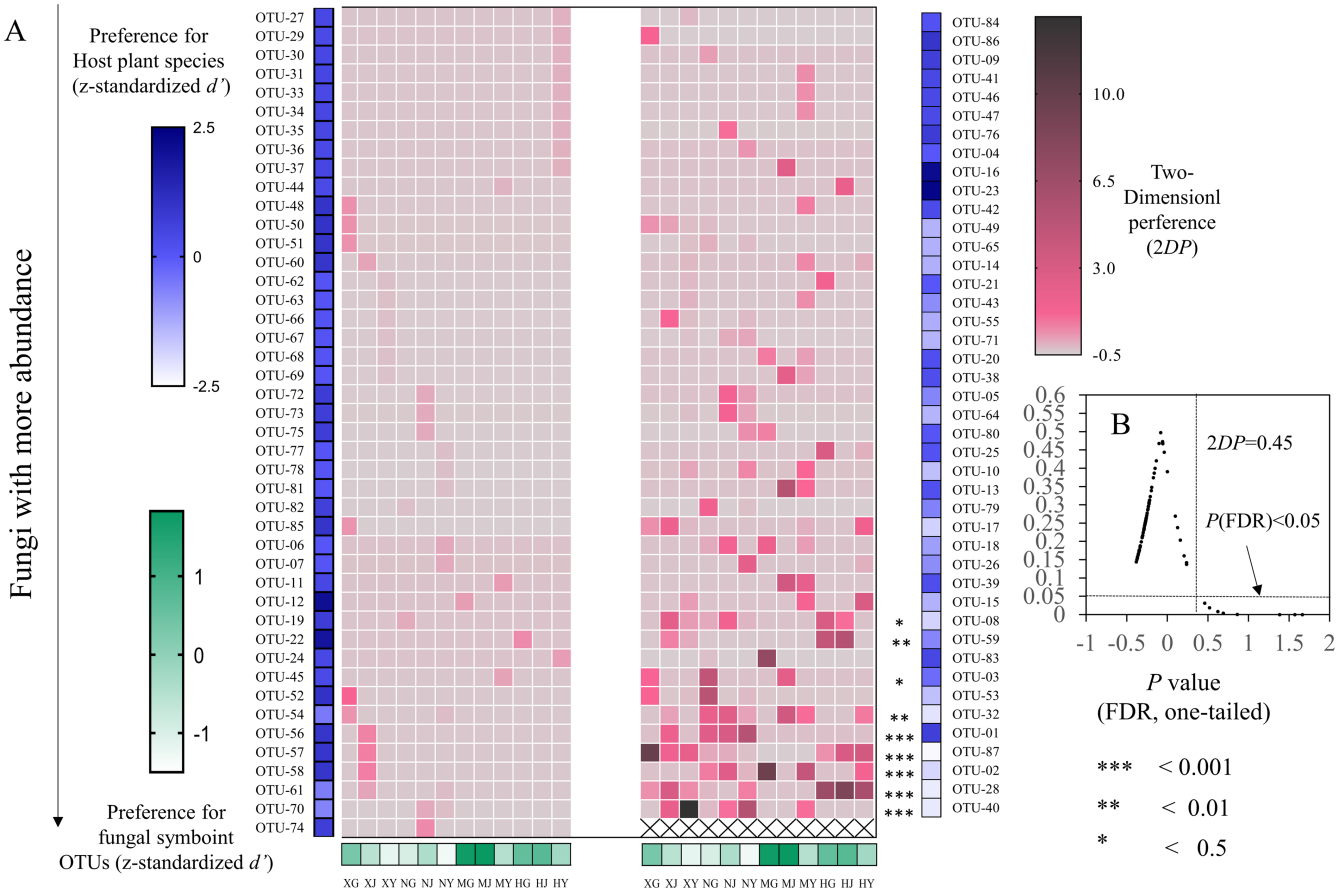
Preferences observed in *G. straminea*–endophytic fungus associations. **(A)** Preference scores, the standardized d’ estimate of preferences for OTUs is shown for *G. straminea* (column). Similarly, the standardized *d*’ estimate of preferences for *G. straminea* is indicated for OTUs (row). Each cell in the matrix indicates a 2*DP* estimate. **(B)** Relationship between 2*DP* and FDR-adjusted *p*-values, 2*DP* values greater than 0.45 represent strong preferences (FDR < 0.05).

The host–fungus association preference analysis revealed that no tissues exhibited a significant preference for endophytic fungi (Figure 10). Of the 87 OTUs of endophytic fungi, 9 exhibited significant preferences for host tissues. It is notable that strong preferences were observed in OTU-01 (*Fusarium fujikuroi*), OTU-28 (*Fusarium*), OTU-02 (*Leptosphaeria*), OTU-40 (*Alternaria*), and OTU-87 (*Penicillium*). In addition, among the match relationships of endophytic fungi and *G. straminea* tissues, 72 out of 1,044 pairs of tissues and fungi exhibited significant preferences, including OTU-02 and root of MY (2DP:9.46) (Figure 10).

### 3.5 LEfSe analysis

**Figure 11:**
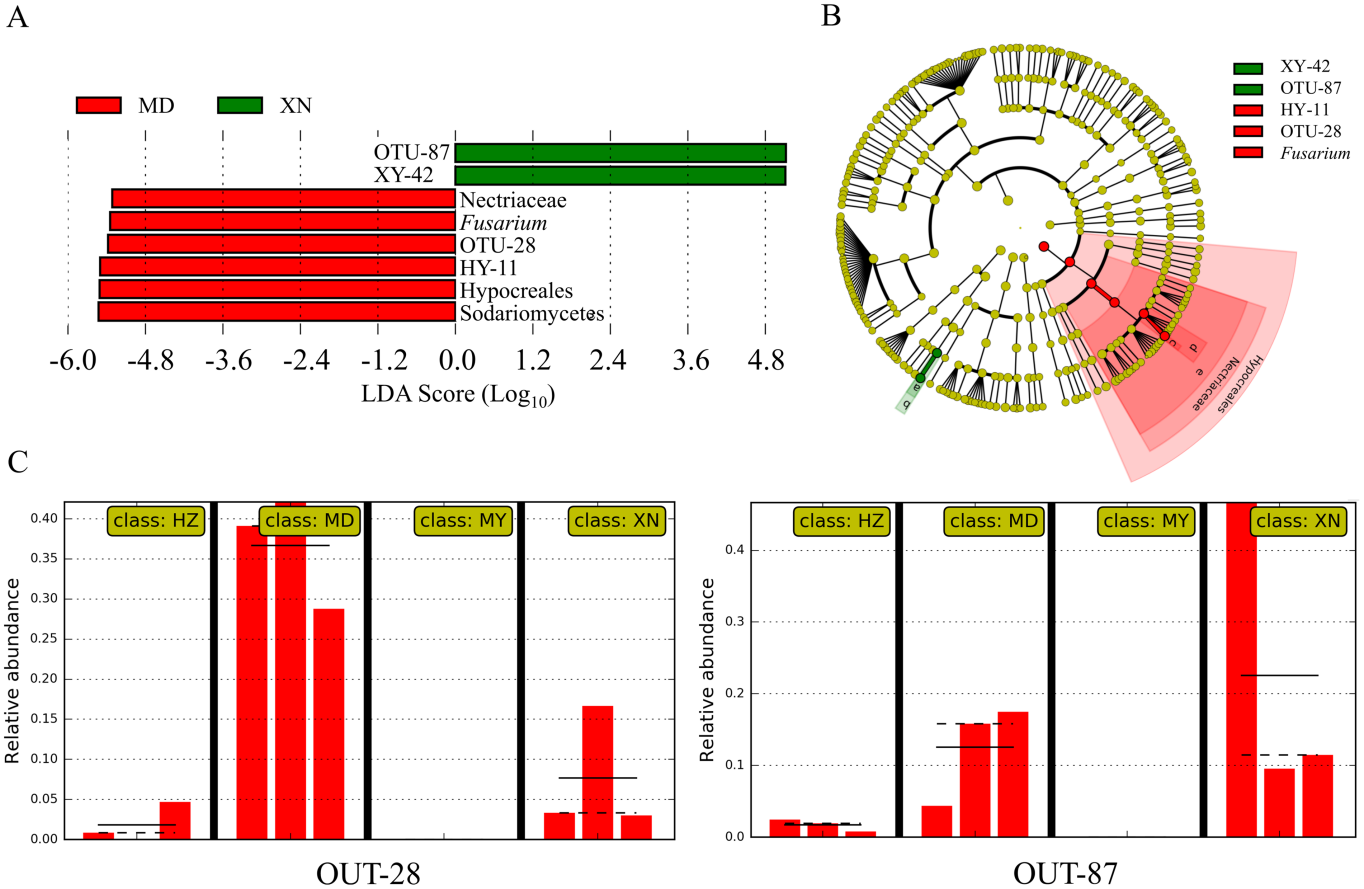
Linear discriminant analysis effects of *G. straminea* from different altitudes. **(A)** LDA scores indicating the effect size of each differentially expressed taxon; **(B)** Cladogram of taxa which were differentially expressed based on LEfSe; **(C)** Relative abundances of endophytic fungi OTU-28 and OTU-87 of *G. straminea* from different altitudes.

To ascertain the species biomarkers of the endophytic fungi, a linear discriminant analysis effect size (LEfSe) was conducted (Figure 11). The eight biomarkers were identified, namely OTU-87 (*Penicillium*), XY-42, *Nectriaceae*, *Fusarium*, OTU-28, HY-11, *Hypocreales,* and *Sodariomucetes* (Figure 11A). The cladogram of taxa analysis demonstrated that OTU-28 and OTU-87 exhibited differential expression (Figure 11B). Of the 87 OTUs, OTU-28 was identified as the biomarker in MD, while OTU-87 was identified as the biomarker of XN (Figure 11C).

### 3.6 Analysis of microbial network topology in *Gentiana straminea*

**Figure 12:**
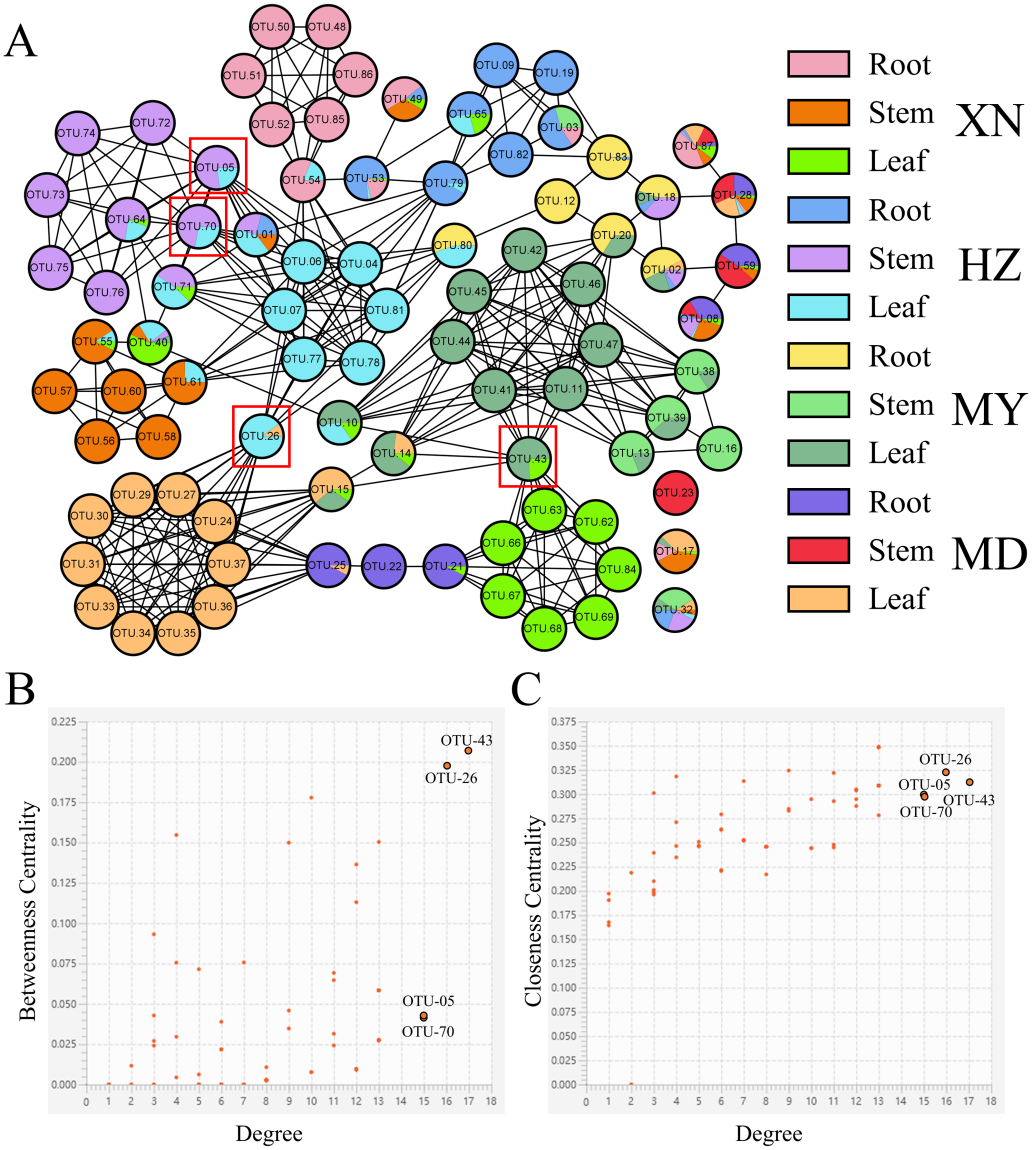
Endophytic fungi co-occurrence network of *G. straminea*. **(A)** Nodes represent OTUs, and edges represent positive (black lines) or negative (red lines) correlations. Node ratio indicates the proportion of tissue mass, according to the legend. Labeled nodes represent the hub OTUs. **(B,C)** Connectivity scores of the nodes, based on degree, betweenness centrality, and closeness centrality, used to identify the hub OTUs.

The distribution of dominant genera exhibited variability among the different samples (Figure 12). The 87 knots constituted the endophytic fungi network topology of *G. straminea*. Each knot displayed exhibited between 1 and 17 sides. In addition to 87 auto-correlations, there were 363 cross-correlations, comprising two negative correlations and 361 positive ones. The results indicated that OTU-43 (*Paraphoma*), OTU-26 (*Cladosporium*), OTU-70 (*Stagonosporopsis*), and OTU-05 (*Fusarium*) were the hub population, with more than 15 lines each, based on the score of direct or indirect centrality traits. In the network, OTU-23 (*Corprinellus*), OTU-17 (*Fusarium*), and OTU-32 (*Colletotrichum*) were the three OTUs, which were found to be independent of one another and to have no relationship with other OTUs (Supplementary Figure 6).

## 4 Discussion

The composition of plant endophytic microorganisms plays a significant role in promoting plant health and adaptation (Yan et al., 2019; Zhang et al., 2020). In this study, 944 strains of endophytic fungi were isolated from different tissues of *G. straminea* at four distinct altitudes on the Qinghai-Tibetan Plateau. The strains were classified into 6 classes, 15 orders, 25 families, 44 genera, and 87 OTUs, representing a richness of fungal species (Figure 1). The study of the colonization rate revealed that the colonization rate of endophytic fungi in the roots was higher in diverse tissues across three locations, with the exception of XN (root<stem< leaf). This can be attributed to the suboptimal disinfection effect resulting from the distinctive root structure of *G. straminea*. Additionally, in comparison with the above-ground parts, the underground parts exhibited a longer survival time (Zhang et al., 2020). The colonization rate of endophytic fungi in the roots of XN was observed to be low, which may be influenced by the local environmental conditions. With regard to altitude, the colonization rate of endophytic fungi in leaves and stems exhibited a decline across the three study areas (XN, HZ, and MY). However, in the MD, the colonization rate was relatively high. Furthermore, no clear trend was observed in the endophytic fungi in the roots along the altitude (Figure 2). In previous studies, it was proposed that the monsoon and the Indian current exert an influence on the plants in the Yellow River region (including MD) (Chen et al., 1999; Zhou et al., 2021). An increase in water richness would result in an increase in the richness of endophytic fungi. In *Eupatorium adenophorum*, the community structure of endophytic fungi in the leaves is more volatile and more susceptible to the surrounding environment (Fang et al., 2019). David et al. (2016) investigated the occurrence and distribution of endophytic fungi in diverse herbaceous plants in coastal ecosystems. Their findings revealed that the separation frequency and abundance of endophytic fungi in leaves are influenced by both the host plant and geographic distance, whereas in roots, these local environmental conditions play a more prominent role in shaping the fungal community. The horizontal transmission of endophytic fungi in plants was predominantly facilitated by wind, water, and soil. In light of these findings, it can be posited that the endophytic fungi present within plant roots are subject to influence from local soil factors. Conversely, the above-ground parts of the plants appear to demonstrate greater susceptibility to external environmental conditions.

In terms of community structure, the results demonstrated that *Ascomycota* is the dominant population, which is in accordance with the findings reported by Egidi et al. (2019). Consequently, the soil fungi richness would contribute to *Ascomycota* in *G. straminea* (Figure 3). The study revealed a significant positive correlation between Ascomycota and loganic acid content, indicating a potential association with secondary metabolites (Fang et al., 2019). At the genus level, *Fusarium* is the most dominant genus in the stems of XN, the stems and leaves of HZ, and MD. *Penicillium* and *Alternaria* are the most dominant in the roots and leaves, respectively, of XN. *Trichoderma* is the most dominant genus of roots and leaves in HZ. *Leptosphaeria* is the most dominant genus in roots and leaves of MY, and the most dominant genus in stems is *Paraphoma* (Figure 5). This finding was consistent with the recent results. In *G. rhodantha*, the endophytic fungus *Fusarium* was the most dominant genus (Zhang et al., 2020), and in *G. rigescens*, the dominant genera were also *Fusarium*, *Aspergillus*, *Penicillium,* and *Alternaria* (David et al., 2016). Furthermore, relevant studies have demonstrated that these genera are ubiquitous in medicinal plants (Egidi et al., 2019). This is attributed to the relatively strong reproductive ability of these strains or the cultivation method that is conducive to their growth. In comparison to other genera, these hyphae demonstrate accelerated growth, an elevated sporulation rate, a greater relative abundance, and a more extensive host range (Egidi et al., 2019; Xu et al., 2020). It is noteworthy that the data set includes a few strains that are relatively uncommon. It is possible that these strains have a low frequency of isolation due to a low parasitic frequency or unsuitable culture conditions. These strains also represent a significant source of novel microbial resources. Cai et al. (2023) have successfully isolated and characterized 10 new xanthone dimers (Subplenones A–J) from the endophytic fungus *Subplenodomus* sp. CPCC 401465, which resides within the *G. straminea*. Additionally, studies have indicated that rare taxa play a significant role in fungal symbiosis networks and ecosystem functions, including crop yield and soil enzyme activity (Xiong et al., 2021). Further investigation is required to ascertain the potential role of these strains in the adaptation of *G. straminea* to alpine regions.

The *α* diversity analysis revealed significant differences between the samples taken from different tissues and altitudes. At the same altitude, the diversity of endophytic fungi in leaves is higher. Xu et al. (2020) employed high-throughput sequencing to investigate the endophytic fungi of *G. rigescens* and discovered that the diversity observed in leaves and flowers is greater than that in roots and stems. As with the colonization rate, the α diversity of root in HZ is the highest, while the MY area is the lowest (Figure 6). This is primarily attributable to the influence of the local environment on the community structure. Furthermore, related studies have also demonstrated that the adjacent plants exert an impact on the community structure of endophytic fungi (Glynou et al., 2016; Vannier et al., 2020). The HZ meadow was situated in close proximity to the forest, whereas the MY meadow was located at a greater distance from the forest. This may be a contributing factor to the observed difference in endophytic fungi. The α diversity of stems exhibited a decline with increasing altitude across all four regions, while the diversity of leaves demonstrated a reduction in the first three regions. However, the PD exhibited an upward trajectory. The study by Rojas-Jimenez et al. (2016) of plant endophytic fungi at varying altitudes within a forest ecosystem concluded that the fungal communities present at different altitudes exhibited distinct compositional characteristics. Furthermore, they noted a decline in diversity with increasing altitude, indicating that the extremes of the environment may exert selective pressure on fungal communities.

The *β* diversity analysis revealed a high degree of similarity between the roots and stems, as well as the leaves and stems. This finding was consistent with that of the previous study (Fang et al., 2019), which proposed that cultured endophytic fungi migrate among the internal tissues of plants and that the stems may serve as a primary conduit for endophytic fungal infection. Nevertheless, the distinction between endophytic fungi in different tissues is minimal, rendering it insufficient for effective differentiation of the various parts (Figure 7). The β diversity of endophytic fungi exhibited variation at different altitudes, with the observed similarity appearing to be more closely associated with geographical location. Despite the considerable altitude difference between XN and MD, the endophytic fungi in the leaves of XN exhibited significant overlap with those in MD, as evidenced by the clustering and NMDS analysis (Figure 8). While altitude does exert an influence on the colonization of endophytic fungi (Figure 9), other factors, including plant community composition, tissue location, and soil changes, have a more pronounced impact, resulting in variations in the distribution of these fungi (Kazenel et al., 2019).

The results of the *G. straminea* endophytic fungi association preference result demonstrated that nine OTUs exhibited a significant preference for host plants. A total of 72 pairs of plants and fungi exhibited a significant preference (Figure 10). Among the identified OTUs, OTU-28 (*Fusarium*) and OTU-87 (*Penicillium*) exhibited a preference for all tissue types within the *G. straminea* populations sampled from MD and XN, respectively (Figure 11). The microbiome hub represents the core of the microbiome and co-occurrence network. The results demonstrated that OTU-43, OTU-26, OTU-70, and OTU-05 constituted the hub population. In a previous study, some endophytic fungi that produce flavonoids were identified (Cheng et al., 2021). The flavonoids have been shown to have powerful antioxidant properties, which can help to clear up free radicals produced during periods of stress. Additionally, the accumulation of gentiopicroside has been found to be more affected by the interaction of microbial communities (Hu et al., 2023). Therefore, these differentially colonized endophytic fungi may play an important role in the authenticity and stress resistance of *G. straminea*. The study revealed that the tissue and altitude exert a considerable influence on the colonization of the endophytic fungi of *G. straminea*. The tissue has an impact on the colonization rate and diversity of endophytic fungi. The changes in climate and space factors coupled with altitude have a significant effect on the composition of endophytic fungi in *G. straminea*, reducing the colonization rate and species abundance in the above-ground part. Additionally, the unique local environment affects the colonization rate and species diversity of underground endophytic fungi.

## 5 Conclusion

This study initially elucidated the diversity profiling of endophytic fungi of *Gentiana straminea* Maxim. under different altitudes. The results demonstrated that the colonization rate and diversity of endophytic fungi were influenced by both tissue type and altitude. The rate of endophytic fungal colonization of tissues was found to be highest in roots, followed by leaves and stems. Furthermore, the *α*-diversity of endophytic fungi among different tissues were leaves>stems>roots, and the PD index in leaves was found to be significantly higher than that in roots. Furthermore, the colonization rate and diversity of endophytic fungi in leaves and stems demonstrated a decline with increasing altitude. The *β*-diversity analysis revealed significant differences in the endophytic fungi of *G. straminea* at varying altitudes. In roots, geographical factors such as latitude and longitude were the primary drivers of diversity, whereas environmental factors, including temperature and precipitation, had a greater influence on endophytic fungi in leaves and stems. In addition, the results of the endophytic fungi association preference, LEfSe, and co-network analysis results indicated that these differential colonized endophytic fungi may play a significant role in the authenticity and stress resistance of *G. straminea*.

## Data Availability

The data presented in the study are deposited in the NCBI repository, accession number PQ415808-PQ416010.

## Supplementary material

SUPPLEMENTARY FIGURE 1 Morphological characteristics of the isolated cultured endophytic fungi of *G.stramine*a.

SUPPLEMENTARY FIGURE 2 **(A)** Venn diagram of endophytic fungi OTUs from tissues of *G. straminea* from different altitudes. **(B)** Rarefaction curves for endophytic fungi from different tissues of *G. straminea* from different altitudes.

SUPPLEMENTARY FIGURE 3 Rarefaction curves for observed endophytic fungi of *G. straminea*.

SUPPLEMENTARY FIGURE 4 Similarity index of endophytic fungi from different tissues of *G. straminea* from different altitudes. (**A**: C_j_ of different altitude; **B**: C_j_ of different tissues).

SUPPLEMENTARY FIGURE 5 UPMGA clustering tree diagram of endophytic fungi from different tissues of *G. straminea* from different altitudes.

SUPPLEMENTARY FIGURE 6 Co-occurrence networks of endophytic fungi at genus leave in the *G. straminea*.
