# Supplementary figures and images for: Distribution and diversity of cultured endophytic fungi in Gentiana straminea Maxim. at different altitudes on the northeastern Qinghai-Tibetan Plateau
Source: Front Microbiol. 2024 Oct 24;15:1466613. doi: 10.3389/fmicb.2024.1466613 (PMC11541051; doi:10.3389/fmicb.2024.1466613)

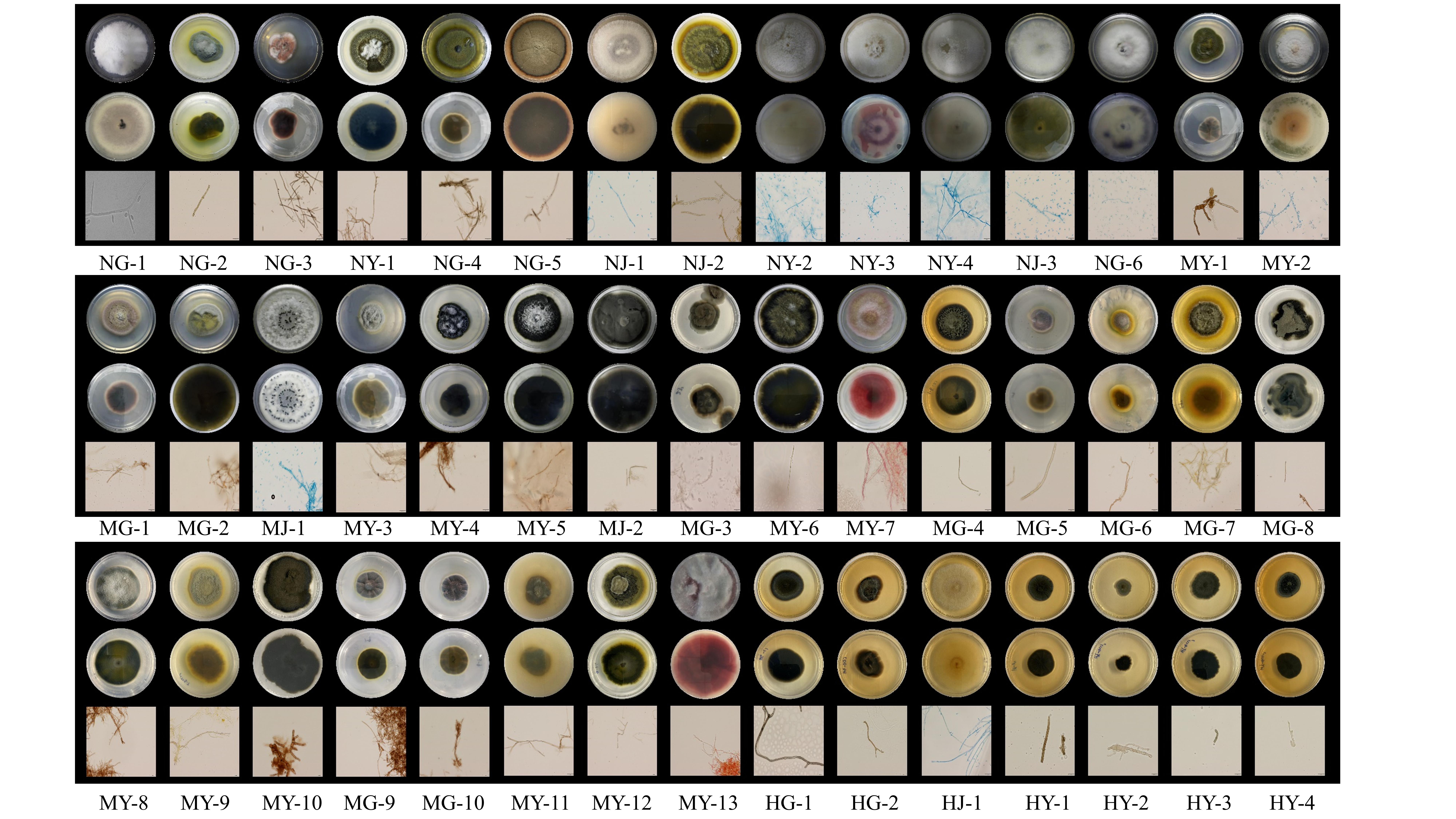

Supplement: SUPPLEMENTARY FIGURE 1 — Morphological characteristics of the isolated cultured endophytic fungi of G.straminea. [file Image_1.TIF]

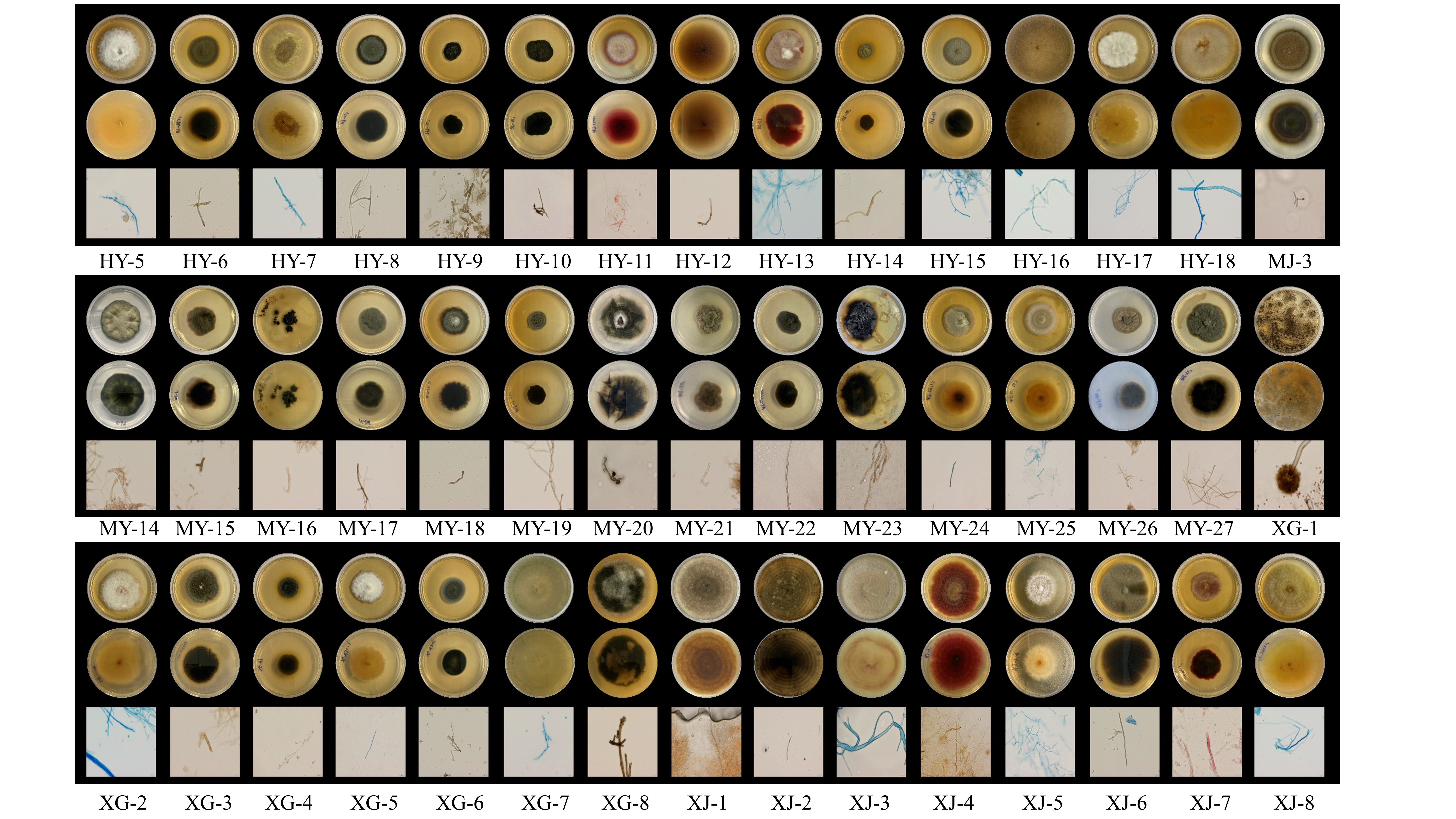

Supplement: SUPPLEMENTARY FIGURE 2 — (A) Venn diagram of endophytic fungi OTUs from tissues of G. straminea from different altitudes. (B) Rarefaction curves for endophytic fungi from different tissues of G. straminea from different altitudes. [file Image_2.TIF]

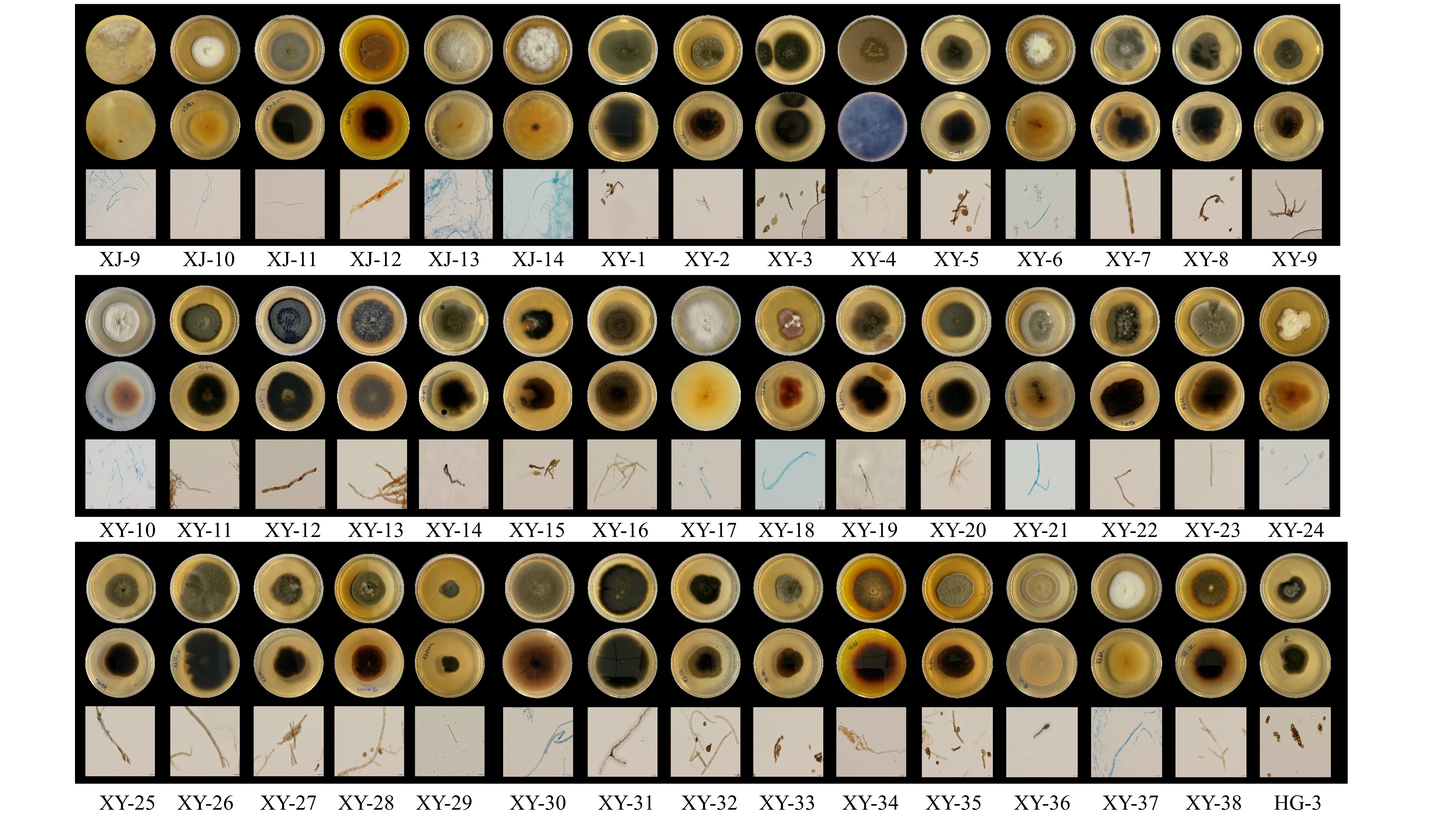

Supplement: SUPPLEMENTARY FIGURE 3 — Rarefaction curves for observed endophytic fungi of G. straminea. [file Image_3.TIF]

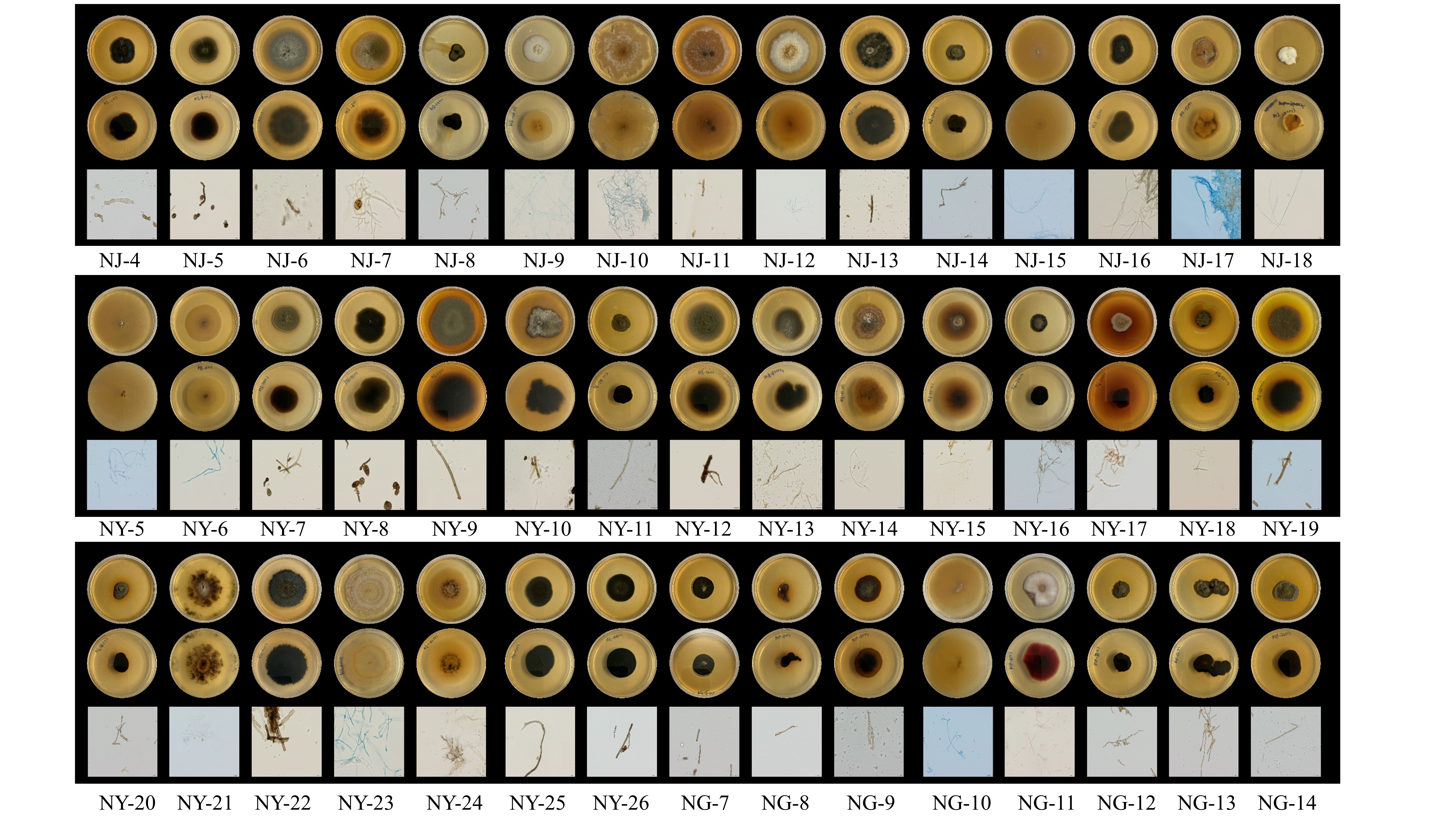

Supplement: SUPPLEMENTARY FIGURE 4 — Similarity index of endophytic fungi from different tissues of G. straminea from different altitudes. (A: Cj of different altitude; B: Cj of different tissues). [file Image_4.TIF]

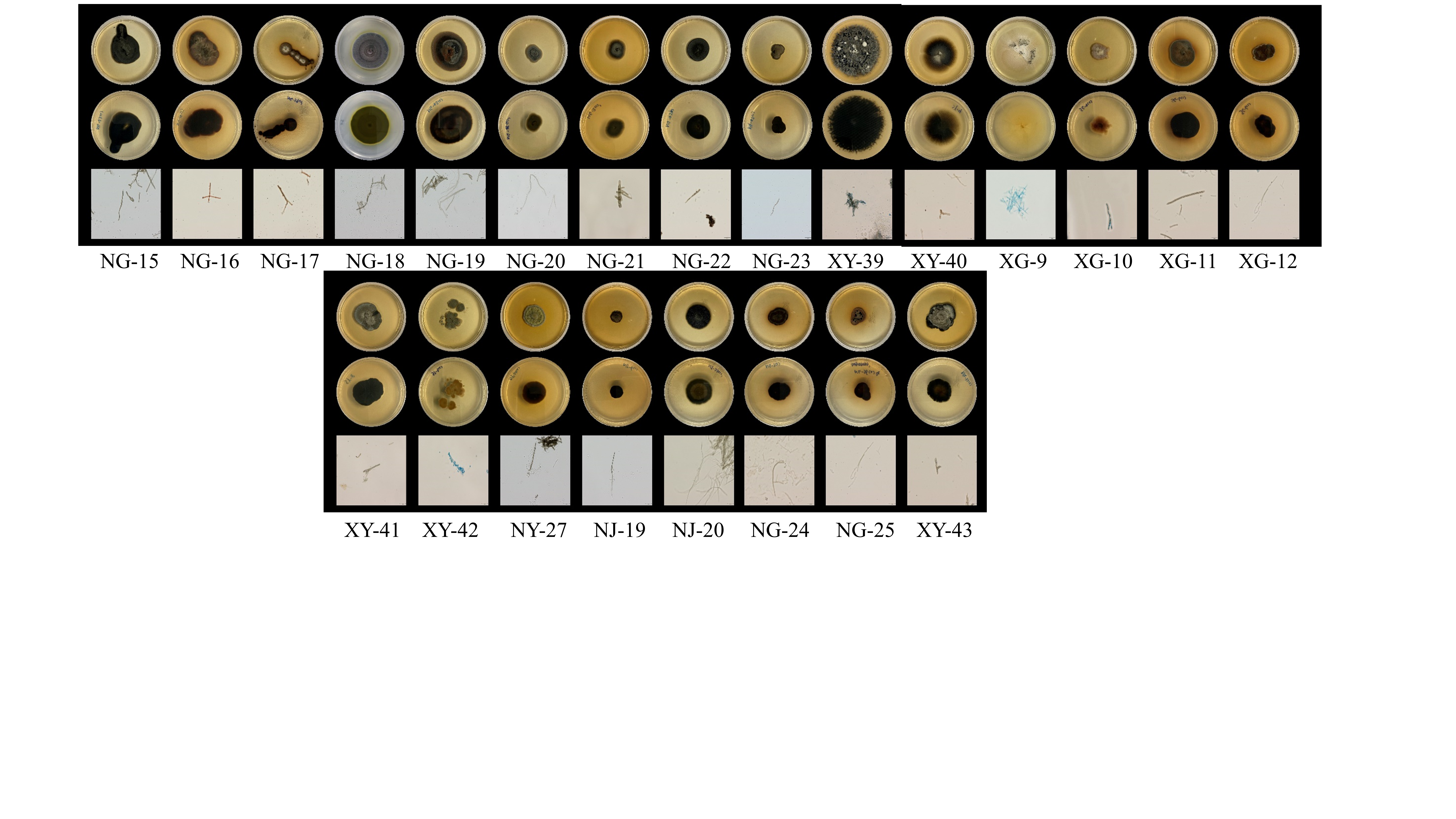

Supplement: SUPPLEMENTARY FIGURE 5 — UPMGA clustering tree diagram of endophytic fungi from different tissues of G. straminea from different altitudes. [file Image_5.TIF]

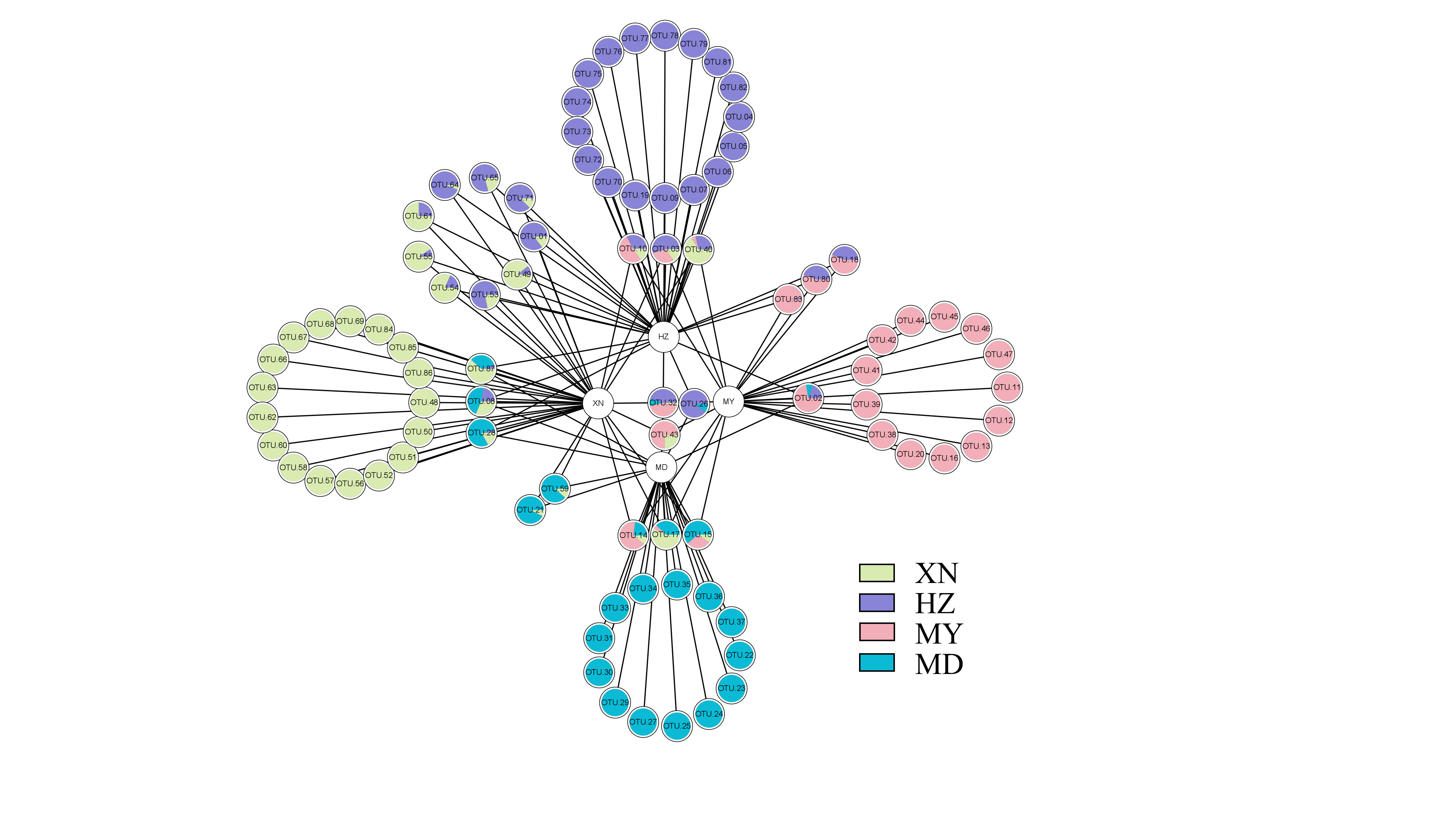

Supplement: SUPPLEMENTARY FIGURE 6 — Co-occurrence networks of endophytic fungi at genus leave in the G. straminea. [file Image_6.TIF]

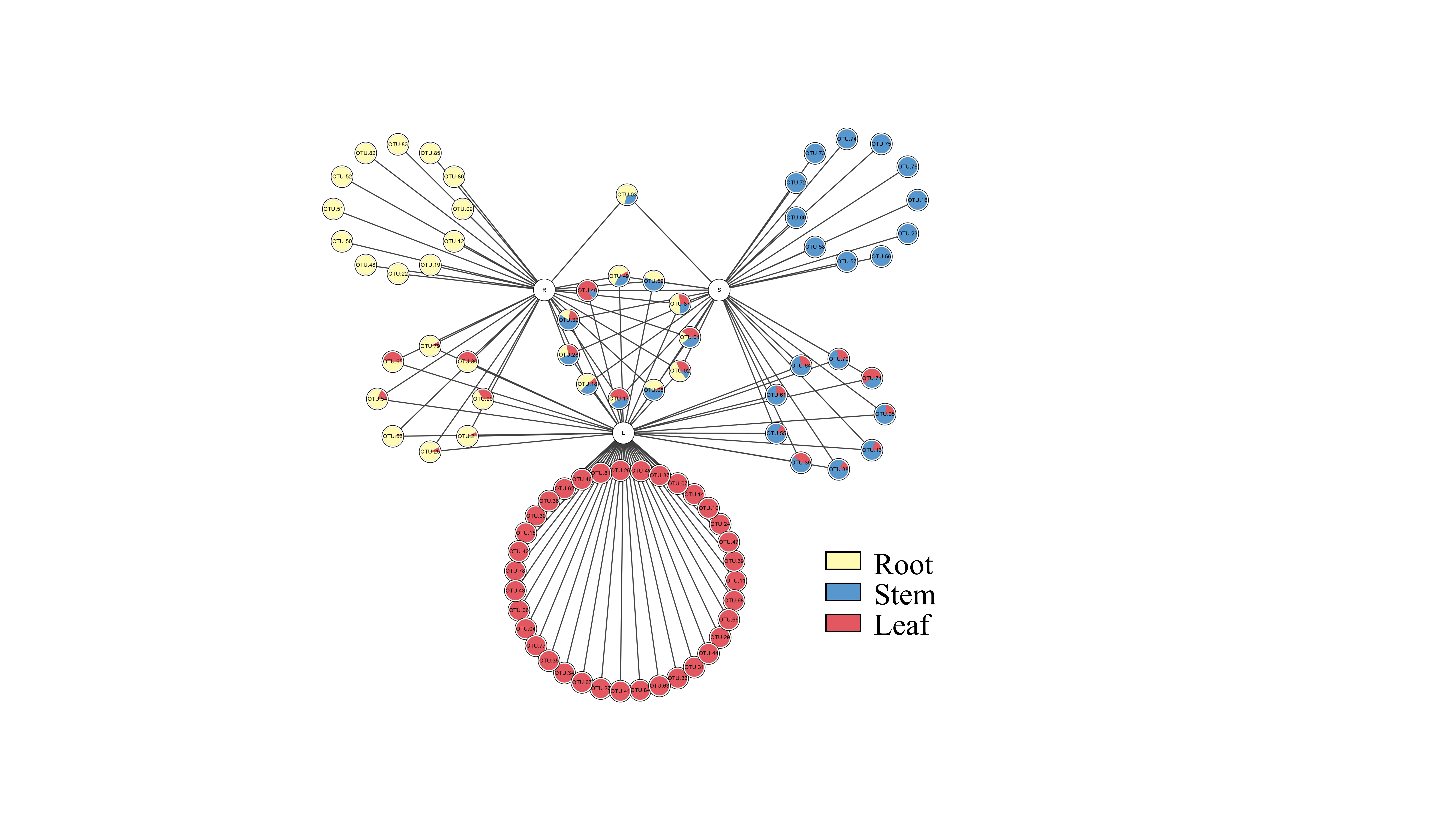

Supplement: Supplementary file 7 [file Image_7.TIF]

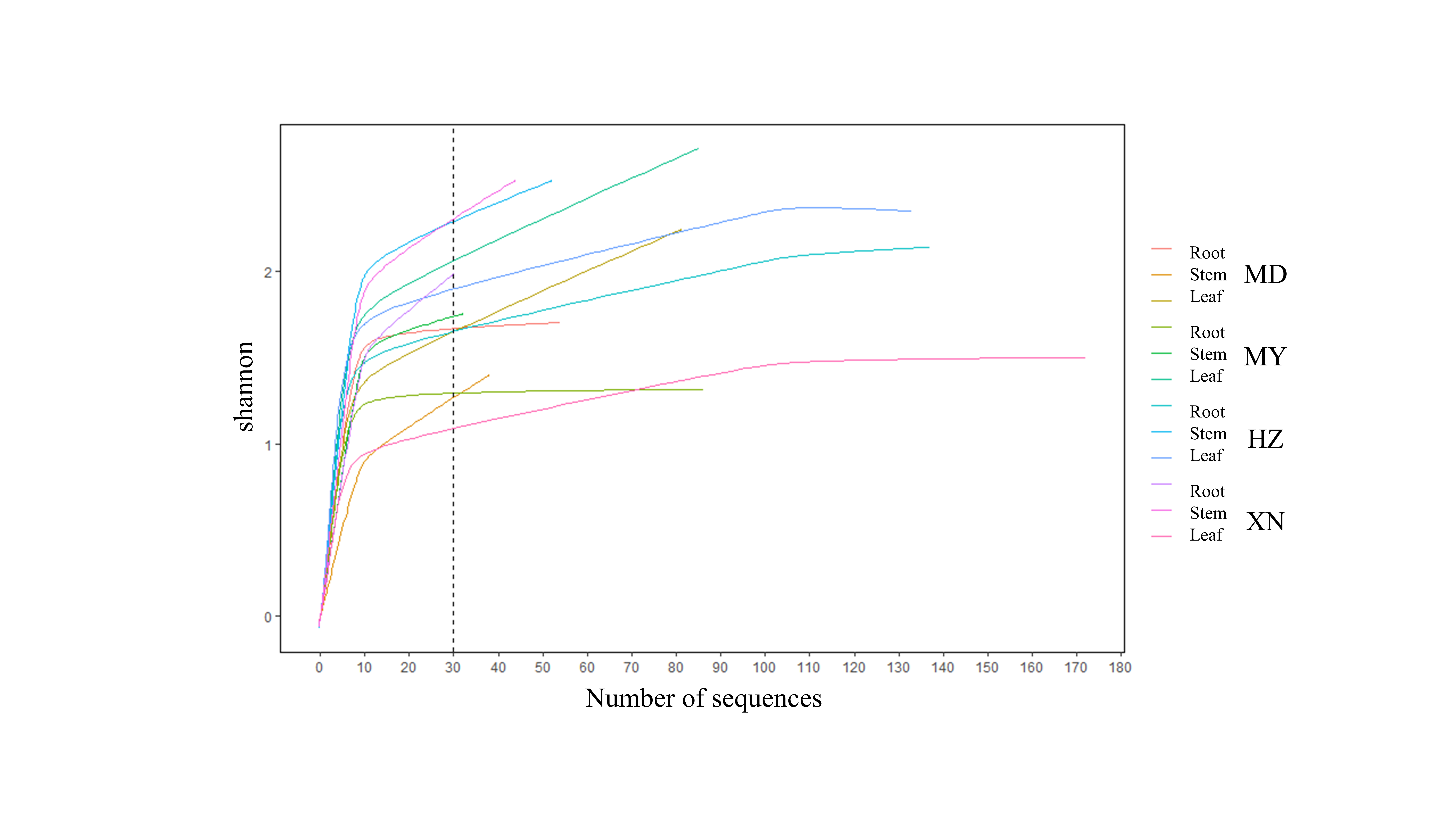

Supplement: Supplementary file 8 [file Image_8.TIF]

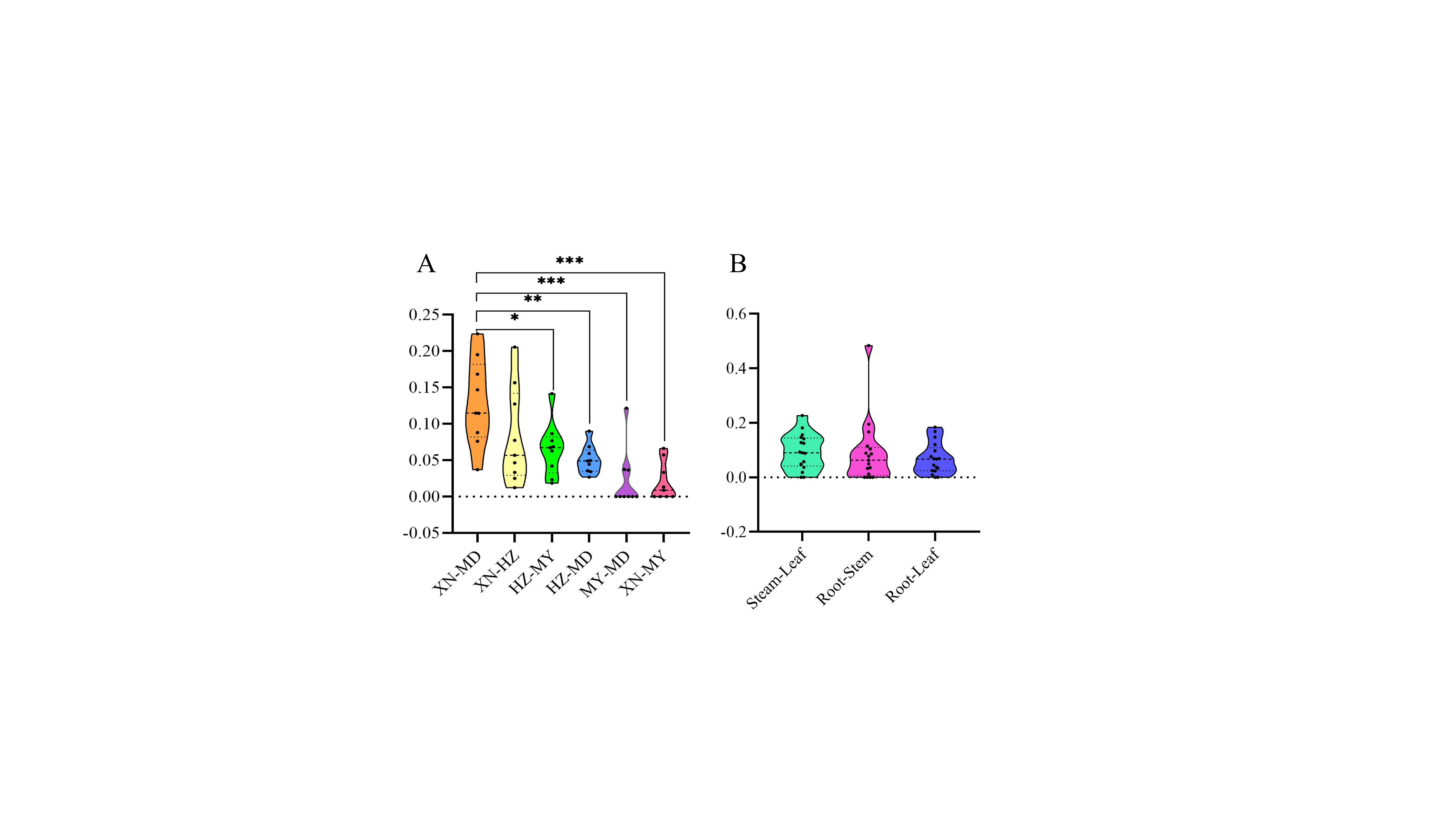

Supplement: Supplementary file 9 [file Image_9.TIF]

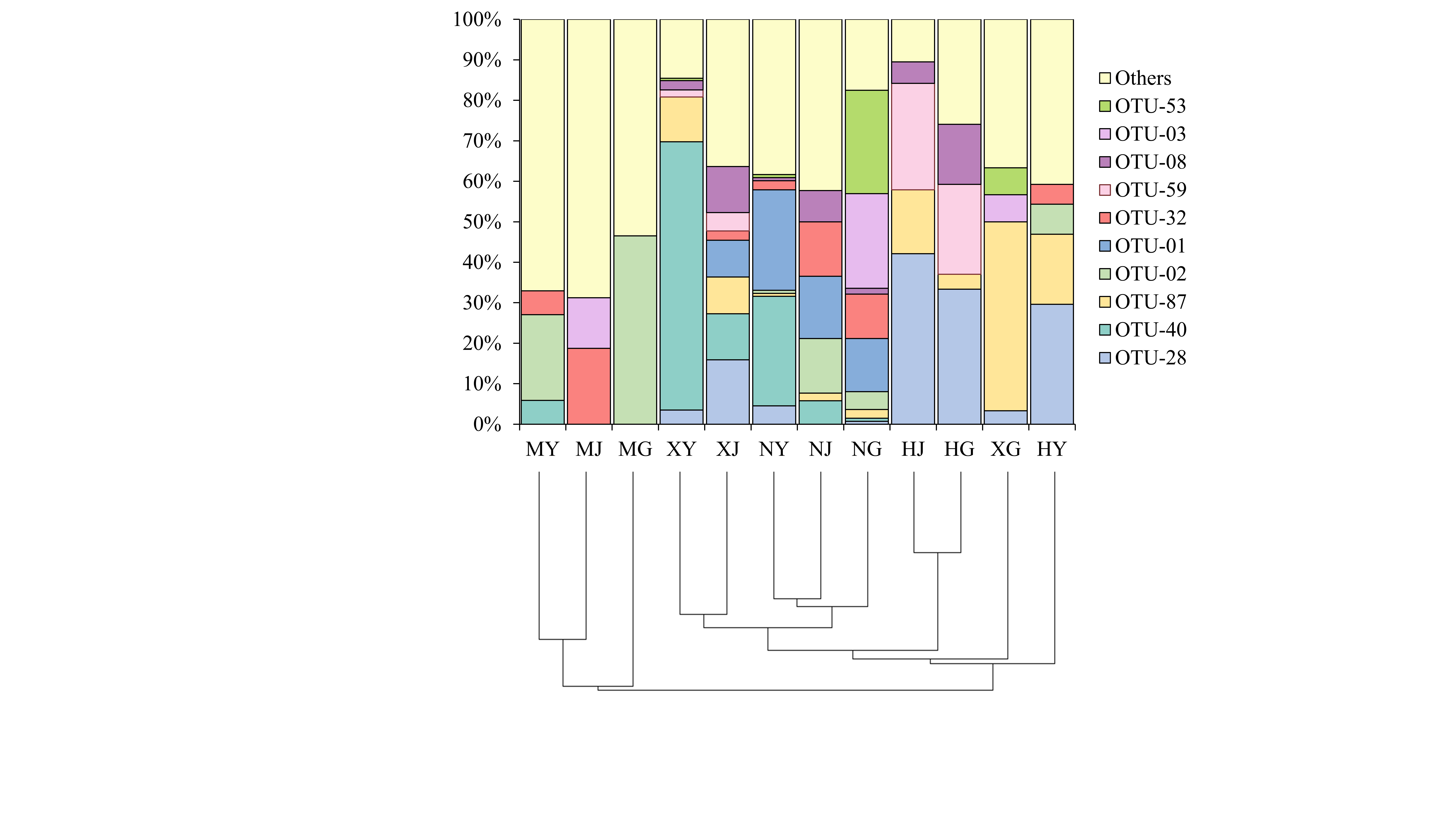

Supplement: Supplementary file 10 [file Image_10.TIF]

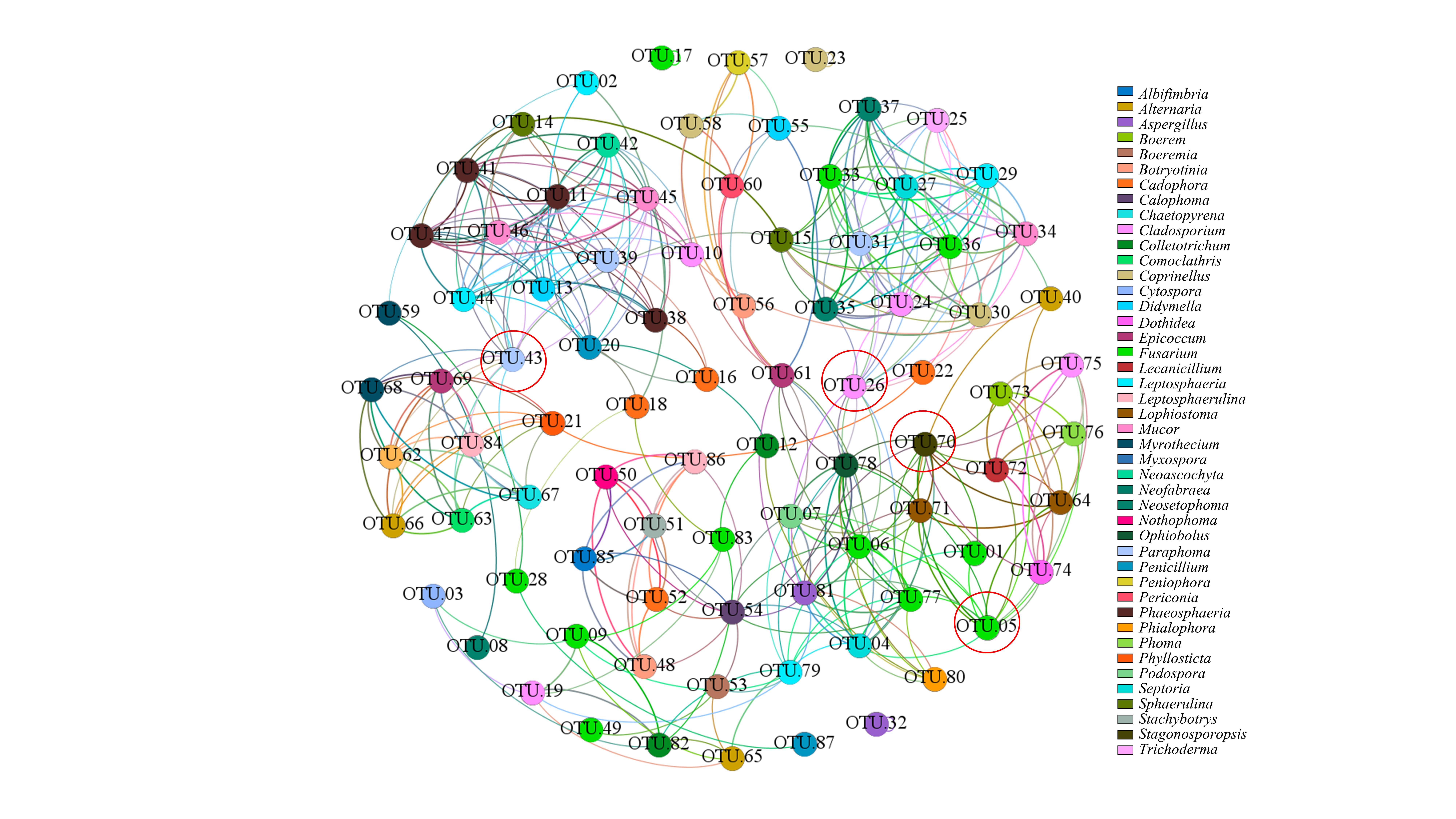

Supplement: Supplementary file 11 [file Image_11.TIF]
